# Construction and function analysis of the LncRNA-miRNA-mRNA competing endogenous RNA network in autoimmune hepatitis

**DOI:** 10.1186/s12920-022-01416-4

**Published:** 2022-12-25

**Authors:** Zhencheng Li, Ying Liu, Yiwen Hou, Zhurong Li, Chen Chen, Huiqin Hao, Yang Liu

**Affiliations:** 1 College of Basic Medical Sciences, Shanxi University of Chinese Medicine, Jinzhong, 030619 People’s Republic of China; 2 Basic Laboratory of Integrated Traditional Chinese and Western Medicine, Shanxi University of Chinese Medicine, Jinzhong, 030619 People’s Republic of China

**Keywords:** Autoimmune hepatitis, Concanavalin A, ceRNA, lncRNA, miRNA, microarray, GM38975, miR-125a-3p, MAPK

## Abstract

**Aims:**

To construct the lncRNA-miRNA-mRNA competing endogenous RNA (ceRNA) network based on our microarray chip data for providing new insights into the pathogenesis of autoimmune hepatitis.

**Methods:**

The ceRNA pairs were obtained by calculating the co-expression relationships among the differentially expressed lncRNAs (DELs), differentially expressed microRNAs (DEMis), and differentially expressed mRNAs (DEMs) with Pearson correlation analysis and hypergeometric distribution. The data of the differentially expressed genes were obtained from our previous studies in the concanavalin A-induced AIH mouse model. The biological functions of the ceRNA network were revealed by carrying out the GO and KEGG enrichment analysis. The expression of some differentially expressed genes constructed in the ceRNA pair was validated, and the correlation to liver injury was analyzed.

**Results:**

The mRNAs constructed in the ceRNA network were most significantly annotated in the GO terms of “inflammatory response” and enriched in “Cytokine-cytokine receptor interaction” and “MAPK signaling pathway”. The differences in the expression of Gm38975, mmu-miR-125a-3p, and Map3k13 between the model group and control group were significant, and the expression of these genes at a transcriptional level was positively or negatively correlated to the activity of ALT and AST as well as the amount of MDA and NO.

**Conclusion:**

Our work is the first in its kind to predict and illustrate the comprehensive lncRNA-miRNA-mRNA ceRNA network associated with the etiopathogenesis of AIH. This study indicates to lay the foundation for revealing the potential roles of ceRNAs in the occurrence of AIH and provide novel treatment targets for this disease.

**Supplementary Information:**

The online version contains supplementary material available at 10.1186/s12920-022-01416-4.

## Research Highlights

- It was the first time to explore the disorder of ceRNA network involved in the pathogenesis of AIH.
- The co-expression relationship between the differentially expressed lncRNAs, miRNAs and mRNAs were predicted.
- The potential biological features and enriched pathways for mRNAs constructed in the ceRNA network, which may be closely associated with the occurrence of AIH, were disclosed.
- Several latent targets, located in the center of the constructed ceRNA network, were provided to investigate the development of this disorder in depth.

**Supplementary Information:**

The online version contains supplementary material available at 10.1186/s12920-022-01416-4.

## Introduction

Since firstly named “active chronic and lupoid hepatitis” by Ian R. Mackay and F. Macfarlane Burnet in 1963, autoimmune hepatitis (AIH) is defined as a type of chronic progressive inflammation of liver parenchyma mediated by autoimmune response at present [1]. It is distinguished in histology by interfacial hepatitis and plasma cell infiltration, as well as in serology by presence of autoantibodies, elevated immunoglobulin G and serum transaminase level [2]. AIH occurs globally in all ages and in all ethnicities with a strong female predominance, and the reported cases are gradually increasing year by year [3]. Although the pathogenesis of AIH referring to immune dysfunction has been basically clarified, which is closely related to the aberrant proliferation and activation of T lymphocytes [4, 5], the detailed mechanism on modulating the expression of genes involved in the development of this disorder is still unclear so far [6].

Competing endogenous RNA (ceRNA) is regarded as the Rosetta stone for interpreting how messenger RNAs (mRNAs) and non-coding RNAs (ncRNAs) “talk’’ to each other via microRNA response elements (MREs). It has greatly expanded the functional genetic information in the human genome and played important roles in pathological conditions [7]. It is believed that two or more RNAs are able to cross-regulate each other indirectly through being targeted by the same microRNA (miRNA), when they share common MREs and compete to bind the same pool of sequences [8]. Such ceRNAs regulate the distribution of miRNAs on their targets, thereby find new significance for ncRNAs and impose an additional level of post-transcriptional regulation [9, 10]. The analyses of ceRNA networks in a broad spectrum of diseases provide new opportunities for interpreting omics data for the field of personalized medicine, including cancer [11, 12], cardiovascular diseases [13, 14], as well as autoimmune diseases [15, 16]. However, ceRNA studies involved in the pathogenesis of AIH have rarely been reported.

Concanavalin A (Con A) can activate T cells, leading to the activation of inflammatory response and the release of inflammatory cytokines. It is generally regarded as a typical inductive agent for establishing the animal model for studying T cell dependent liver injury and simulating pathological changes in patients with AIH [17, 18]. Hence, the long non-coding RNA (lncRNA)-miRNA-mRNA ceRNA network in an AIH mouse model induced by Con A, which was regarded as the most important AIH research model [19–22], was constructed for the first time based on the screened the differentially expressed lncRNAs (DELs), miRNAs (DEMis) and mRNAs (DEMs) using microarray chip from our previous studies [23, 24]. The functional assignment of ceRNA network were systematically analyzed with bioinformatic methods herein, in order not only to provide a new insight into the etiopathogenesis of AIH, but also to offer state-of-the-art therapeutic strategy for this worldwide hepatitis.

## Materials and methods

### Regents and chemicals

Con A was bought from Solarbio Science & Technology Co., Ltd. (Beijing, China, catalog number: C8110). Malondialdehyde (MDA) assay kit (TBA method), Alanine aminotransferase (ALT) Assay Kit, and Aspartate aminotransferase (AST) Assay Kit were purchased from Nanjing Jiancheng Bioengineering Institute (Nanjing, China, catalog number: A003-1-2, C009-2-1 and C010-2-1). Total Nitric Oxide (NO) Assay Kit was gotten from Beyotime Biotechnology (Shanghai, China, catalog number: S0021S). Chloral hydrate, UNlQ-10 Column Total RNA Isolation Kit and 2X SYBR Abstart Master Mix was purchased from Sangon Biotech Co., Ltd. (Shanghai, China, catalog number: A600288, B511321 and B110032). The Total Protein Extraction Kit (BOSTER Biological & Technology Co. Ltd., China, catalog number: AR0146), SDS–polyacrylamide gel electrophoresis (SDS-PAGE, Beyotime Biological Technology Co. Ltd., China, catalog number: P0012A), PVDF membranes (Millipore, USA, catalog number: R1CB73920), High-sig ECL Western Blotting Substrate (Tanon Science & Technology Co., Ltd., China, catalog number:180-501), Anti-p38 Rabbit pab, Mapk3k13 Rabbit pab, HRP-conjugated Goat Anti-Rabbit IgG (Additional file 1: Table S1) were used to detect the expression of target protein.

### Construction of ceRNA network

**Fig. 1:**
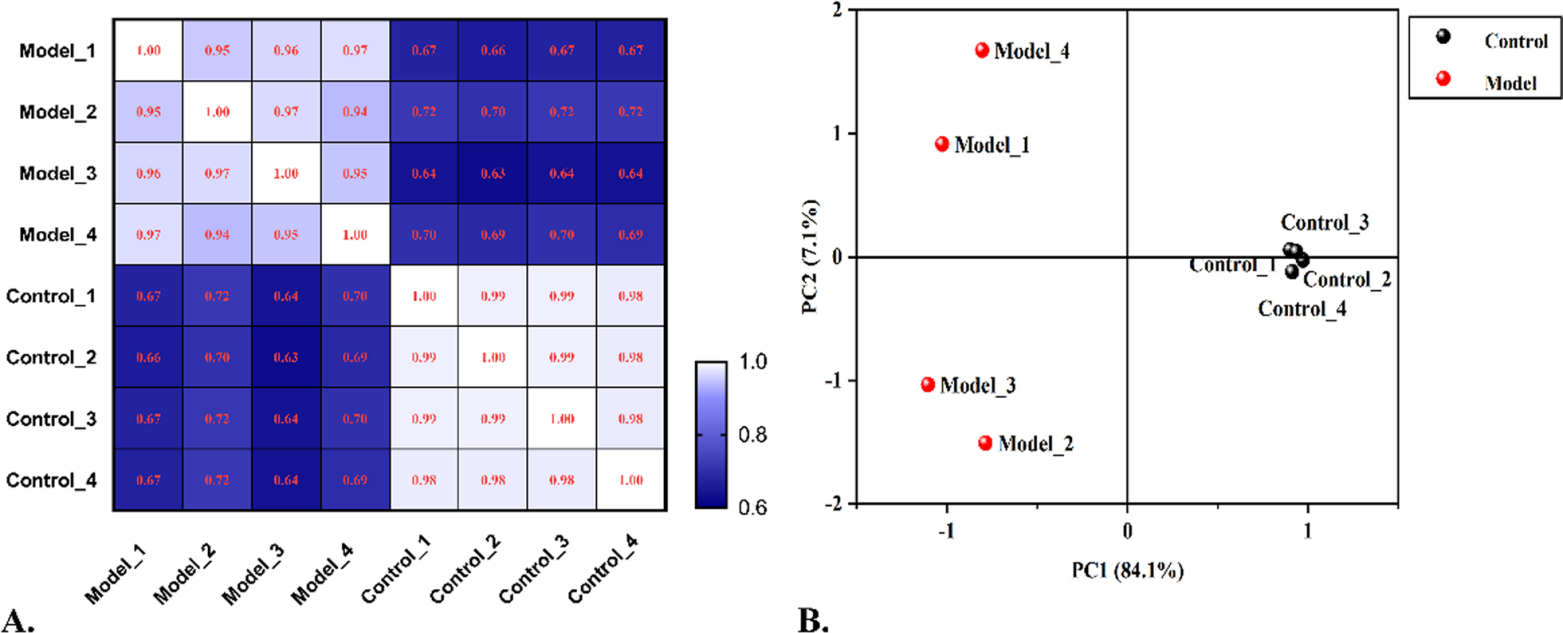
Evaluation of the screened data. **A** Pearson correlation analysis. Each box represented one sample in different groups, and the decimal in the box was the correlation coefficient between different samples. The larger the number, the lighter the color of each box, which meant the higher the relevance. **B** Results of PCA. The red and black dots signified the individuals in the model and control group, respectively. The shorter the distance between samples in the same group and the more significant the separation between samples in different groups, while the more the reproducibility of the data and rationality of sample selection

**Fig. 2:**
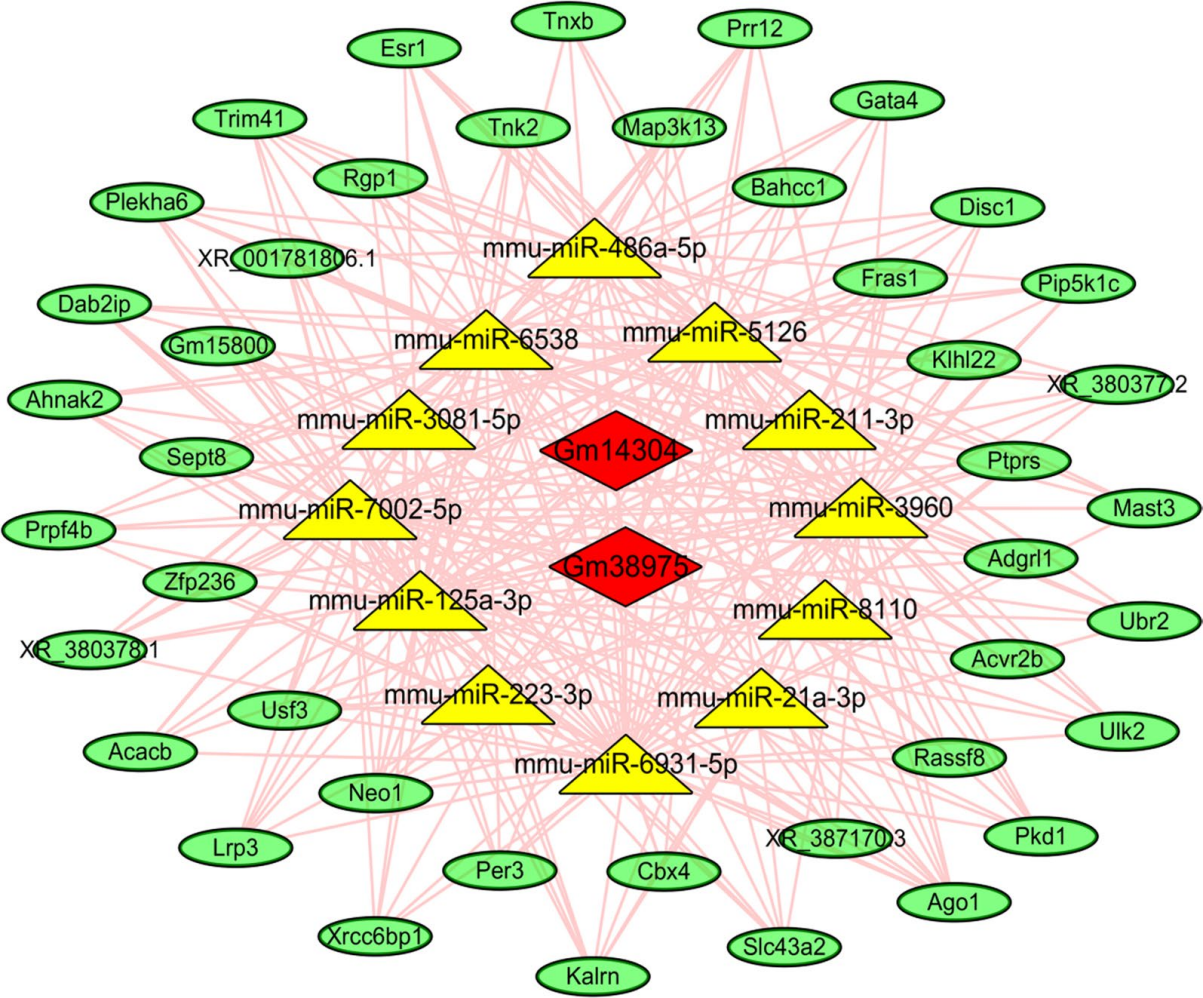
CeRNA network consists of the top 200 mRNA-miRNA-lncRNA pairs. Red diamond, yellow triangle, and green ellipse nodes indicated the DELncs, DEMis, and DEMs, respectively. The pink edges meant the regulatory relationships between different genes

**Fig. 3:**
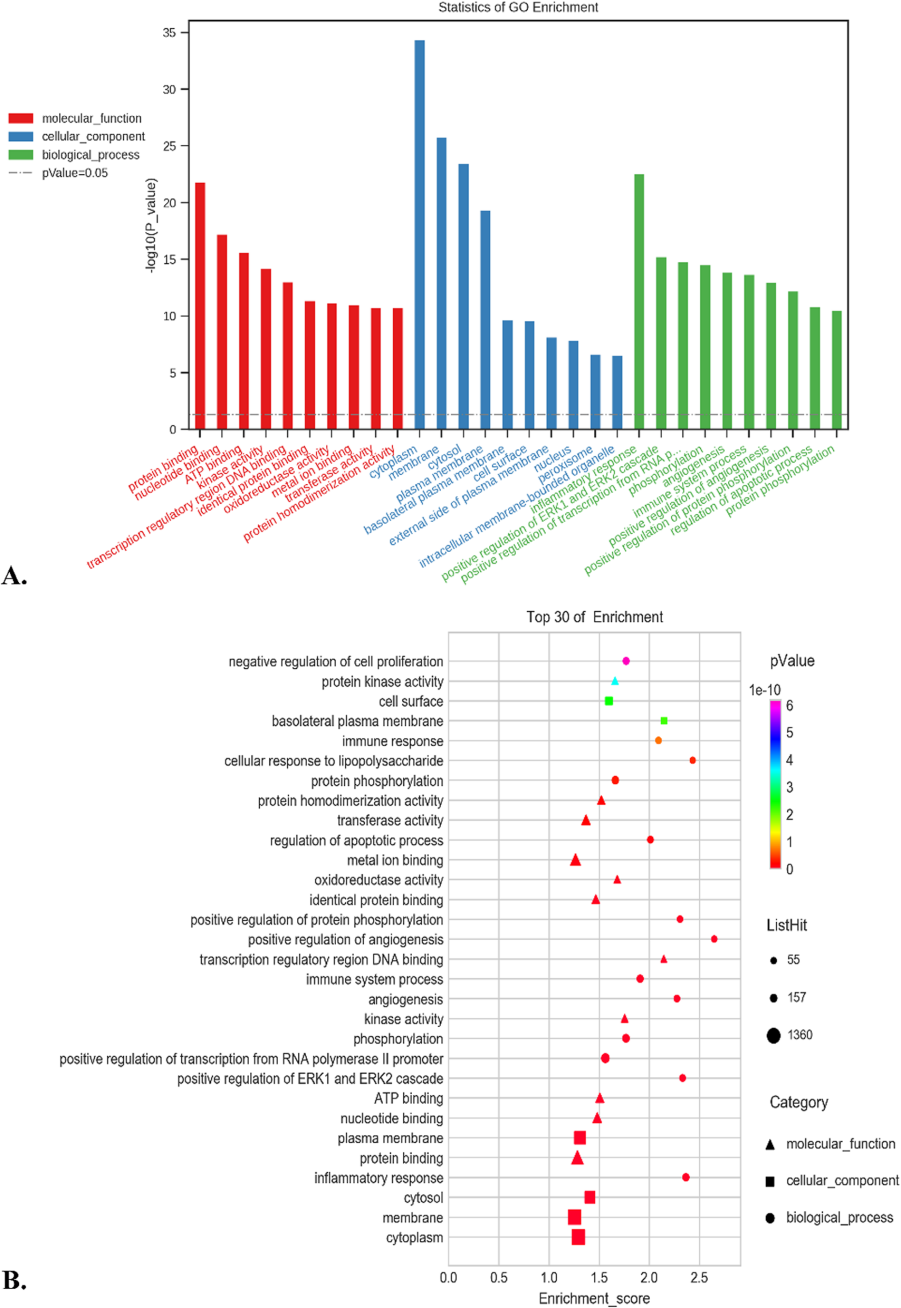
GO enrichment analysis. GO enrichment analysis of DEMs was performed based on hypergeometric distribution using the R Programming Language software. **A** Top 10 GO terms in different categories in ascending order of *P* value. **B** Top 30 of all GO terms in ascending order of *P* value

The data of DELs, DEMis and DEMs in the Con A-induced AIH model were collected from our other experiments, including 1,161 DELs (608 up- and 553 down- regulated), 49 DEMis (31 up- and 18 down-regulated) and 11,512 DEMs (5,189 up- and 6,323 down-regulated) [23, 24]. In order to evaluate the reproducibility of the data, correlation analysis between different samples were implemented with *Pearson* correlation and Principal component analysis (PCA) (see Fig. 1). Thereafter, the miRNA-lncRNA, miRNA-mRNA and lncRNA-mRNA co-expression relationships were firstly analyzed with *Pearson* correlation analysis according to the threshold of |*Pearson* correlation coefficient (PCC)|≥ 0.80 and *P* value ≤ 0.05, and the regulatory relationships between miRNA-lncRNA and miRNA-mRNA were predicted in accordance with default parameter of “miranda V3.3a” software (Max Score ≥ 140 and Total Energy ≤ − 2.0) (see Fig. 2). The total scores of each pair of miRNA-lncRNA or miRNA-mRNA were calculated with the “miranda V3.3a” software, and the MREs of each pair of miRNA-lncRNA or miRNA-mRNA were predicted and counted according to the seed region, which is the most evolutionarily conserved fragment on miRNA and usually completely complementary to the target site on mRNA or lncRNA. Then, the ceRNA score of each predicted pair were also calculated with the ceRNA MuTATE method [25]. Combining with the probability of sharing same miRNAs among ceRNA pairs calculated by hypergeometric distribution algorithm [26], the ceRNA relation pairs with high reliability were obtained. Finally, the corrected ceRNA relation pairs were acquired by taking the intersection of analysis of “ceRNA scores” and results of mRNA-lncRNA co-expression relationship. A simplified ceRNA network consisting of the top 200 lncRNA-miRNA-mRNA pairs (in the descending order of “ceRNA Score”) was constructed with Cytoscape software (version 3.7.2, http://cytoscape.org/), so as to visualize the underlying regulatory function of these differently expressed genes (DEGs) (see Fig. 3).

### Function prediction of DMEs in the constructed ceRNA network

**Fig. 4:**
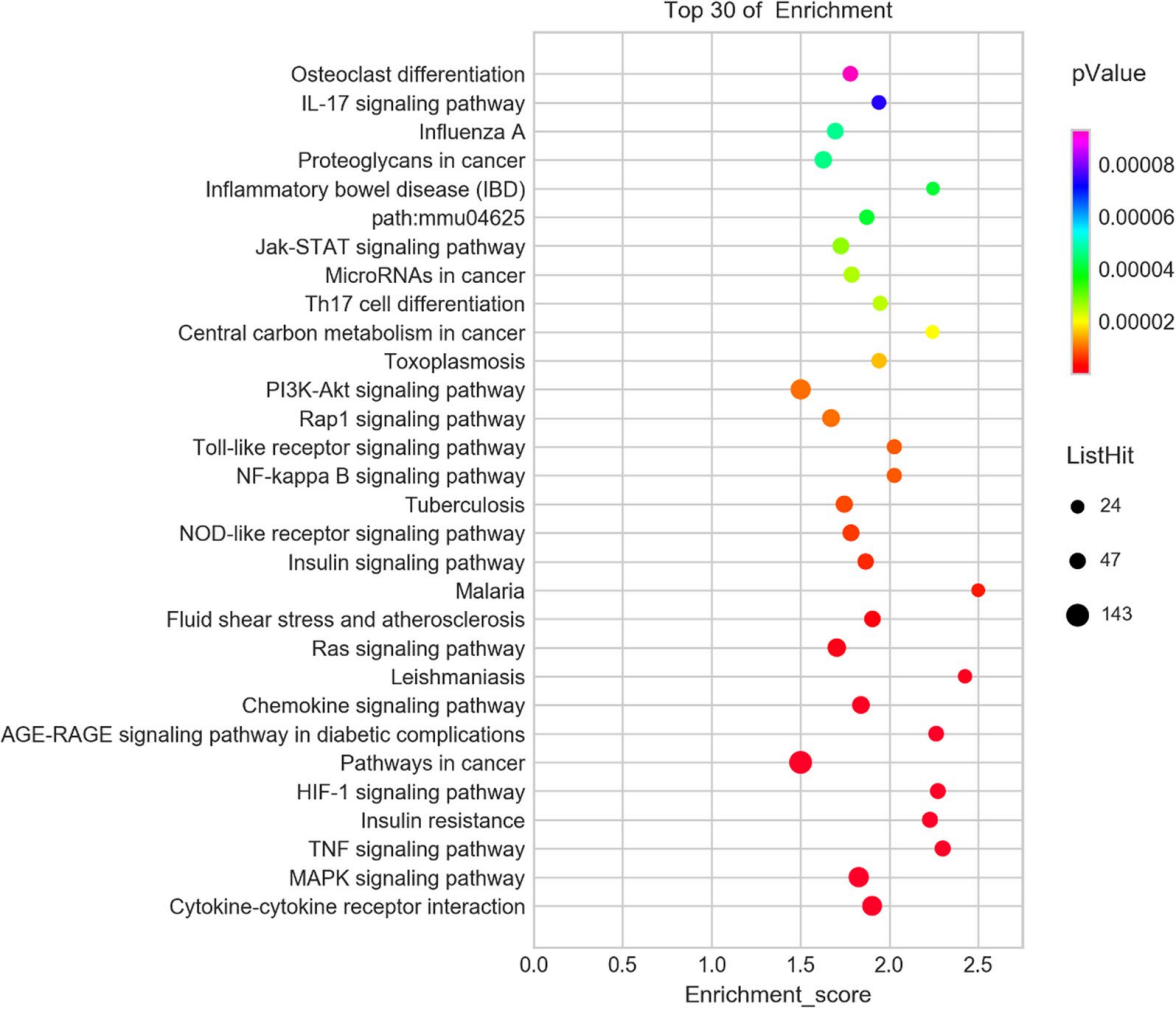
KEGG pathway analysis. Hypergeometric distribution was implemented in the KEGG enrichment analysis via the R Programming Language software, and the top 30 pathways in ascending order of *P-value* were shown

**Fig. 5:**
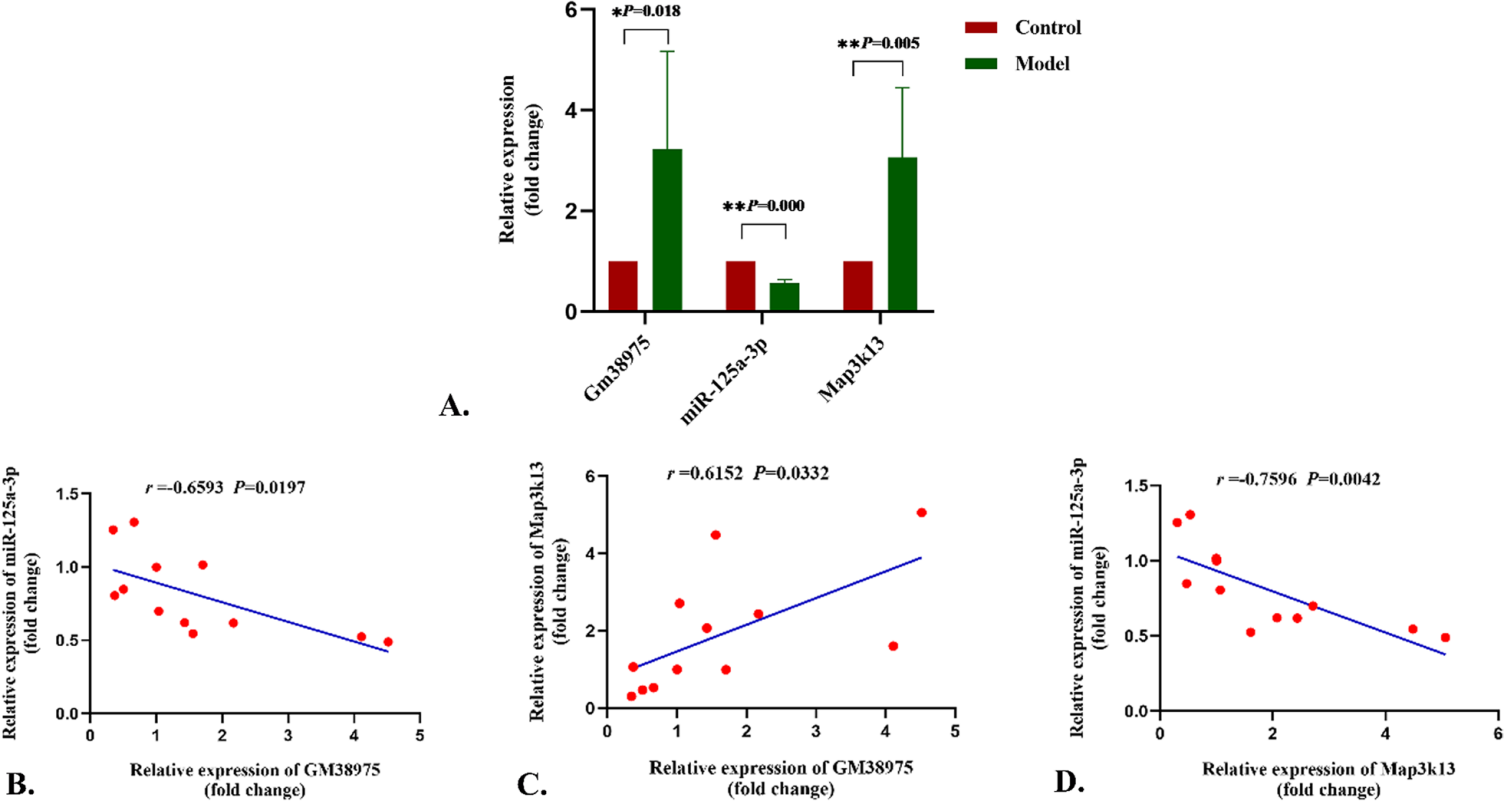
Relative expression of the selected differentially expressed genes. **A** The relative expression levels of *Gm38975*, *mmu-miR-125a-3p,* and *Map3k13* in the model group compared with the control group. The data were normalized to *U6* and shown with the mean ± SD (error bars), then statistically analyzed by Student’s *t*-test. **B**–**D** The correlation between *mmu-miR-125a-3p* transcriptional expression levels and *Gm38975* or *Map3k13* transcriptional expression levels was measured by *Spearman* correlation analysis. *P* < 0.05 was considered statistically significant (*P < 0.05, ** *P* < 0.01)

Gene ontology (GO) enrichment analysis was carried out with the method of hypergeometric distribution algorithm using the *R Programming Language software* (Package ggplot2) (http://www.r-project.org/) to have a deep insight into the characteristics of DEMs involved in the ceRNA network (see Fig. 4) [27, 28]. *Fisher*'s exact test was used to calculate the enrichment significance of each GO term in categories of cellular components (CC), molecular functions (MF) and biological processes (BP) [29]. The false discovery rate (FDR), applied to the multiple testing corrections of raw *P-*value, was set as the cutoff for selecting significantly enriched functional GO terms (FDR ≤ 0.05). According to the Kyoto Encyclopedia of Genes and Genomes (KEGG) enrichment analysis (Release 85.0, January 1, 2018, https://www.genome.jp/kegg/), the underlying biological activities of these DEMs were also calculated with the method of hypergeometric distribution algorithm via the R Programming Language software (see Fig. 5) [27, 28]. The recommended FDR ≤ 0.05 was the threshold for genes which were eligible to be annotated in the pathways, and lower *P* value represented higher correlation between pathways and DEMs.

### Animal experiments

In order to verify the results of ceRNA network prediction, and to search reliable new targets for further studies, twelve specific pathogens free (SPF) male C57BL/6 mice (25–28 g) were selected to perform the animal experiment. All the mice were obtained from Vital River Laboratory Animal Technology Co., Ltd. (Beijing, China), and randomly divided into model group (n = 6) and control group (n = 6). They were housed under controlled temperature (21–24 °C) and humidity (40–60%), 12 h dark/light cycle and feeding ad libitum. The establishment of AIH model and samples collection were in accordance with our former experiments [23, 24]. Hepatic tissues of each mouse were harvested by surgery under low temperature (performing on ice) and sterile conditions, and stored in a deepfreezer (below − 80 °C). The blood samples were collected using a tube and then centrifuged. The separated serum was used for analyzing the activity of hepatic enzymes and the amounts of oxidative stress products (MDA and NO). All animal studies were performed in compliance with the guidelines of ARRIVE (https://arriveguidelines.org). Meanwhile, the animal experiments were approved by the Ethics Committee of Shanxi University of Chinese Medicine (Permit Number: 2019LL41), and complied with the rules of National Institutes of Health guide for the care and use of laboratory animals (NIH Publications No. 8023, revised 1978) and the institutional rules of animal experimentation approved by the Experimental Animal Ethics Committee of Shanxi University of Chinese Medicine.

### RNA isolation and real-time PCR

Some of the differently expressed genes constructed in the concise ceRNA network were chosen for validation, including GM38975 (Accession number: XR_001780316.1), mmu-miR-125a-3p (Accession number: MIMAT0004528) and Map3k13 (Accession number: NM_172821.3). The microarray analysis features of these DEGs were list in Additional file 1: Table S2. Part of the collected hepatic tissue samples were immediately homogenized with UNlQ-10 Column Total RNA Isolation Kit for extracting total RNA. The integrity of the extracted RNA was assessed with Agilent Bioanalyzer 2100 (Agilent Technologies, USA). The primers of these genes were synthesized by Sangon Biotech Co., Ltd. (Shanghai, China) based on the sequences obtained from the National Center of Biotechnology Information (NCBI) database, and the sequences were itemized in Additional file 1: Table S3. All the selected aberrantly expressed genes were amplified with one-step SYBR Green assay in a Step One PLUS System (ABI, Foster, CA, USA). Expression of these genes were normalized to *U6* or *GAPDH*, and were calculated with the 2^−ΔΔCt^ method [30]. Fold change (FC) represented the relative transcriptional expression level of the selected genes.

### Western blotting analysis

In order to verify the relationship between the expression of Map3k13 and the pathogenesis of AIH at the translational level, the expression of Map3k13 and p38 (the downstream effector protein of Map3k13) were detected with western-blot assay. Total lysates of hepatic tissues were obtained using the Total Protein Extraction Kit according to the product specification. Proteins were separated by SDS-PAGE and transferred to PVDF membranes. The target bands were cropped according to the different molecular weights of the protein prior to hybridization with antibodies. Membranes were immunoblotted with primary antibodies (Anti-Mapk3k13 Rabbit pab) at 4 °C overnight. After incubation with HRP-conjugated Goat Anti-Rabbit IgG at room temperature for two hours, the blots were detected with the Azure Imaging Systems C300 (Azure Biosystems, CA, USA) after incubating with the High-sig ECL Western Blotting Substrate. The loading control for the western blotting was β-Actin, and the densities of the bands were assessed and normalized to the β-Actin signals.

### Serum biochemical analysis

The severity of liver injury can be determined by the activity of serum ALT and AST. The blood samples obtained from all the mice were kept at room temperature for 30 min. Then, serum was collected after centrifugation at 1000*g* for 15 min. The activity of serum ALT and AST were measured with Alanine aminotransferase Assay Kit and Aspartate aminotransferase Assay Kit according to the manufacture's instructions.

### Measurement of MDA and NO

Parts of the liver tissues (100 mg) collected from all the mice were homogenized. The amounts of MDA and NO were determined in line with the manufacture's instructions of Malondialdehyde assay kit (TBA method) and Total Nitric Oxide Assay Kit.

### Statistical analysis

All statistical analyses in this study were performed using SPSS 25.0 software (SPSS Inc., Chicago, IL, USA). All experimental data were expressed as the means ± standard deviation (SD), and statistically analyzed by Student’s *t*-test. The correlation between expression of differential expression genes and hepatic injury were also calculated by *Spearman’s* correlation analysis method. Statistical significance was considered as *P* value < 0.05 from a two-tailed test.

## Results

### Co-expression analysis

As shown in Fig. 1A, each box represented the correlativity between different samples (the data in the box was the coefficient of association). The lighter the color of box and the larger the number, the higher the correlation. The normalized data of each sample in control group was with a very high relevance, and the same for the model group. According to the result of PCA in Fig. 1B, we also found that the distance between samples in the same group was relatively close, and the samples in different groups were relatively discrete, i.e., individuals in model group flocked together and separated significantly from the control group. It was indicated that reproducibility of the data and rationality of sample selection satisfied the conditions for further analysis.

A total of 62,754 miRNA-mRNA pairs, 2,972 miRNA-lncRNA pairs and 395 8730 mRNA-lncRNA co-expression pairs with positive correlation were gotten in this experiment, and the top 10 pairs of miRNA-mRNA, miRNA-lncRNA and mRNA-lncRNA in descending order of “Total Score” or “PCC” were enumerated in Tables 1, 2and 3. The higher the “Total Score” or “PCC”, the more significant the co-expressive relationship. The top 5 pairs of miRNA-mRNA and miRNA-lncRNA in descending order of “Total Score” were exhibited in Additional file 1: Fig. S1, and the MREs were clearly labeled on the mRNA or lncRNA sequences.

**Table 1:** Top 10 miRNA-lncRNA pairs in descending order of Total Score

miRNA	lncRNA	r	*P* value	microRNA response element	Total score
mmu-miR-6931-5p	XR_868659.2	− 0.921441255	1.14E−03	11	1645
mmu-miR-877-3p	XR_874399.2	− 0.828603429	1.10E−02	11	1617
mmu-miR-6931-5p	XR_868658.2	− 0.876762747	4.26E−03	10	1498
mmu-miR-6931-5p	XR_882206.1	− 0.803718936	1.62E−02	9	1426
mmu-miR-6931-5p	XR_875698.2	− 0.930024003	8.12E−04	9	1357
mmu-miR-7005-5p	XR_868659.2	− 0.927772486	8.92E−04	9	1313
mmu-miR-3960	NR_038049.1	− 0.814906061	1.37E−02	8	1240
mmu-miR-3960	NR_038048.1	− 0.834675307	9.94E−03	8	1240
mmu-miR-3081-5p	XR_379583.3	− 0.876548296	4.28E−03	8	1157
mmu-miR-6931-5p	XR_376934.3	− 0.81135297	1.45E−02	7	1081

**Table 2:** Top 10 miRNA-mRNA pairs in descending order of Total Score

miRNA	mRNA	r	*P* value	microRNA response element	Total Score
mmu-miR-1927	*XM_017319372.1*	− 0.984300367	9.56E−06	48	6816
mmu-miR-3058-5p	*XM_017318325.1*	− 0.833434446	1.02E−02	23	3631
mmu-miR-6931-5p	*NM_001040398.2*	− 0.885133958	3.47E−03	23	3565
mmu-miR-6931-5p	*NM_175022.2*	− 0.84944667	7.60E−03	19	2828
mmu-miR-193b-3p	*NM_144848.2*	− 0.831252664	1.05E−02	18	2565
mmu-miR-6931-5p	*NM_001005475.2*	− 0.816768065	1.33E−02	16	2447
mmu-miR-6931-5p	*XM_006531124.2*	− 0.837480877	9.47E−03	15	2270
mmu-miR-22-5p	*NM_144848.2*	− 0.801094057	1.69E−02	15	2178
mmu-miR-3960	*XM_017315330.1*	− 0.929577698	8.28E−04	15	2153
mmu-miR-877-3p	*XM_006537476.2*	− 0.868403244	5.15E−03	15	2218

**Table 3:** Top 10 mRNA-lncRNA pairs in descending order of Pearson correlation coefficient

lncRNA id	lncRNA symbol	mRNA id	Gene symbol	Pearson correlation coefficient	*P*-value
XR_879821.1	GM42074	*NM_001082552.2*	TRIM21	0.999736712	4.56E−11
XR_001782924.1	LOC108168645	*XM_006520578.3*	SLC11A2	0.999639794	1.17E−10
XR_874481.1	GM35164	*XM_006532716.2*	SREBF1	0.999538999	2.45E−10
XR_878666.1	LOC102634900	*XM_006539980.2*	TARM1	0.999503873	3.05E−10
XR_871273.1	GM40579	*NM_021704.3*	CXCL12	0.999484476	3.42E−10
NR_130109.1	FENDRR	*NM_176835.2*	DNAJC22	0.999457612	3.99E−10
XR_872804.1	GM40438	*NM_029880.3*	PTGR2	0.999397854	5.46E−10
XR_375276.3	GM32287	*NM_001033767.3*	GM4951	0.999350462	6.85E−10
XR_871774.2	1110002J07RIK	*XM_006495719.2*	IL1R1	0.999331009	7.48E−10
NR_033450.1	SERPINA3H	*NM_001033335.3*	SERPINA3F	0.999266756	9.85E−10

### Construction of ceRNA network

In the light of set threshold of *P* value ≤ 0.05, 658,316 ceRNA pairs were predicted. The top 10 pairs in descending order of “ceRNA Score” were particularized in Table 4. The lower the *P* value, the more significant the correlation between the mRNA or lncRNA and their shared miRNAs. Based on the intersection of “ceRNA score” and the results of mRNA-lncRNA co-expression relationship analysis, 545,204 filtered ceRNA pairs were gotten. The ceRNA network consisting of the top 200 mRNA-miRNA-lncRNA pairs (in the descending order of “ceRNA Score”) was constructed with Cytoscape software.

**Table 4:** Top 10 ceRNA pairs in descending order of ceRNA Score

mRNA	lncRNA	ceRNA Score		*P* value	Shared miRNA
*NM_013630.2*	XR_001780316.1	0.3626	2.90E−03		7002-5p;125a-3p; 6931-5p; 3960; 5126; 486a-5p; 6538
*NM_001317174.1*	XR_001780316.1	0.3626	2.90E−03		7002-5p; 125a-3p; 6931-5p; 3960; 5126; 486a-5p; 6538
*XM_006532819.3*	XR_001780316.1	0.3469	2.00E−03		7002-5p;125a-3p; 6931-5p; 3960; 5126; 486a-5p; 6538
*XM_017317338.1*	XR_001780316.1	0.3469	2.00E−03		7002-5p; 125a-3p; 6931-5p; 3960; 5126; 486a-5p; 6538
*XM_006510846.3*	XR_001780316.1	0.3469	2.00E−03		7002-5p; 125a-3p; 6931-5p; 3960; 5126; 486a-5p; 6538
*NM_146078.3*	XR_001780316.1	0.3311	1.40E−03		7002-5p; 125a-3p; 6931-5p; 3960; 5126; 486a-5p; 6538
*XM_017317340.1*	XR_001780316.1	0.3311	1.40E−03		7002-5p; 125a-3p; 6931-5p; 3960; 5126; 486a-5p; 6538
*XR_380378.1*	XR_001780316.1	0.3311	1.40E−03		7002-5p; 125a-3p; 6931-5p; 3960; 5126; 486a-5p; 6538
*XM_006510843.3*	XR_001780316.1	0.321	3.30E−02		7002-5p; 125a-3p; 6931-5p; 3960; 5126; 6538
*XM_006531124.2*	XR_001780316.1	0.3153	9.00E−04		7002-5p; 125a-3p; 6931-5p; 3960; 5126; 486a-5p; 6538

The duplicated “mmu-miR” in the name of each Shared miRNA was deleted in order to make the table a little more concise

### Functional prediction of DEMs involved in ceRNA network

With the recommended threshold of FDR ≤ 0.05, 3 263, 3 272 and 3 241 DEMs were annotated in 266 BP terms, 57 CC terms and 89 MF terms, respectively. The GO terms with the lowest *P* value in the categories of BP, CC and MF were “inflammatory response” (GO:0006954), “cytoplasm” (GO:0005737) and “protein binding” (GO:0005515), severally. The top 10 GO terms in different categories and the top 30 of all GO terms (in ascending order of *P* value) were shown in Fig. 3, in order to help to further explore the potential roles of these DEMs in the pathogenesis of AIH.

For the result of KEGG pathway analysis, there were 1426 DEMs annotated in 105 signaling pathways. Ranked in ascending order of *P* value, the top 30 pathways with the lowest *P* values were displayed in Fig. 4, and the top 3 pathways were “Cytokine-cytokine receptor interaction” (path: mmu04060), “MAPK signaling pathway” (path: mmu04010) and “TNF signaling pathway” (path: mmu04668). These results can provide a better understanding of the underlying regulatory function of these DEMs participated in the onset of AIH.

### qRT-PCR validation

As lay out in Fig. 5A, the differences of the selected genes expression between model group and control group were statistically significant, including GM38975 (FC = 3.37 ± 2.38, *P* < 0.05), mmu-miR-125a-3p (FC = 0.68 ± 0.06, *P* < 0.01), Map3k13 (FC = 3.06 ± 1.39, *P* < 0.01), and the PCR results were in accordance with the microarray analysis. The negative correlations between mmu-miR-125a-3p expression and GM38975 or Map3k13 expression, as well as the positive correlation between GM38975 expression and Map3k13 expression at transcriptional level were confirmed by the *Spearman* correlation coefficients (Fig. 5B–D). These data support that the high GM38975 or Map3k13 transcriptional level expression in hepatic tissues is associated with liver injury and negatively associated with transcriptional level expression of mmu-miR-125a-3p, which can provide truthful biological markers for further study.

### Western blotting analysis

**Fig. 6:**
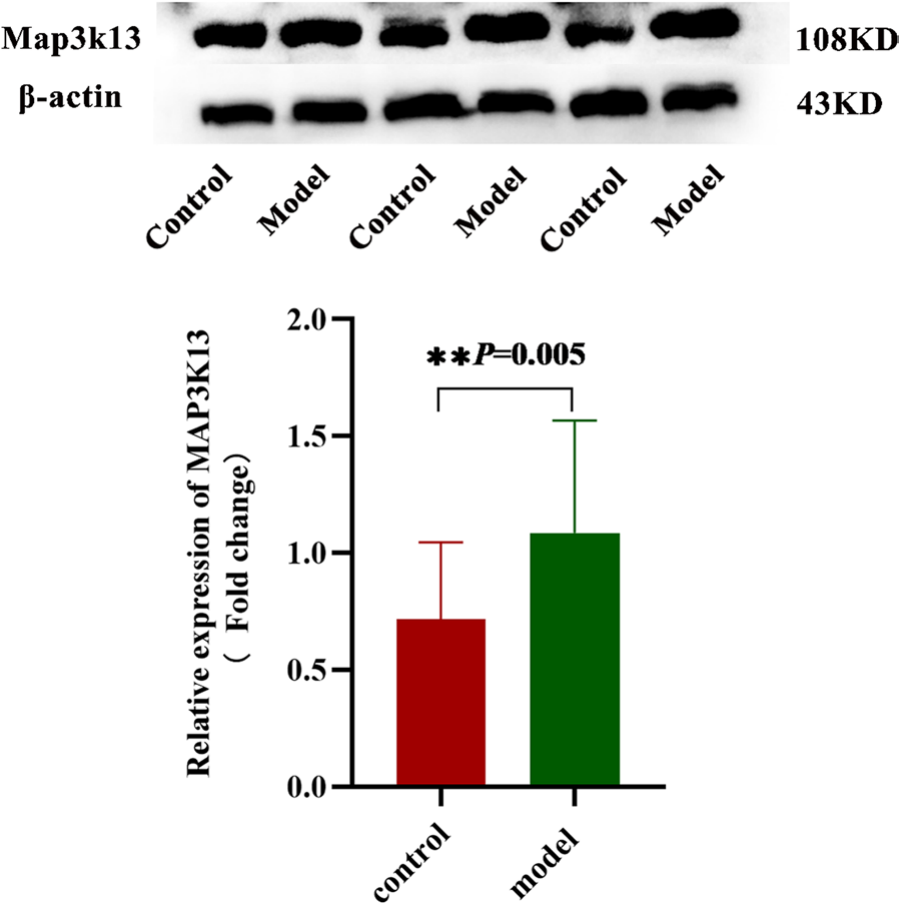
Relative expression of the Map3k13 protein. Compared with the control group, the hepatic Map3k13 protein expression level was significantly elevated in the model group *P* < 0.05 was considered statistically significant. The target bands were cropped according to the different molecular weights of the protein prior to hybridization with antibodies (*P < 0.05, ** *P* < 0.01). All the blots were exhibited in Additional file 1: Fig. S2

Compared with the control group, the hepatic Map3k13 protein expression level was significantly elevated in model group (Fig. 6) (*P* < 0.01). These data also indicate that the aberrant translation expression of Map3k13 is related with the hepatic damage.

### Activity of serum ALT and AST

**Fig. 7:**
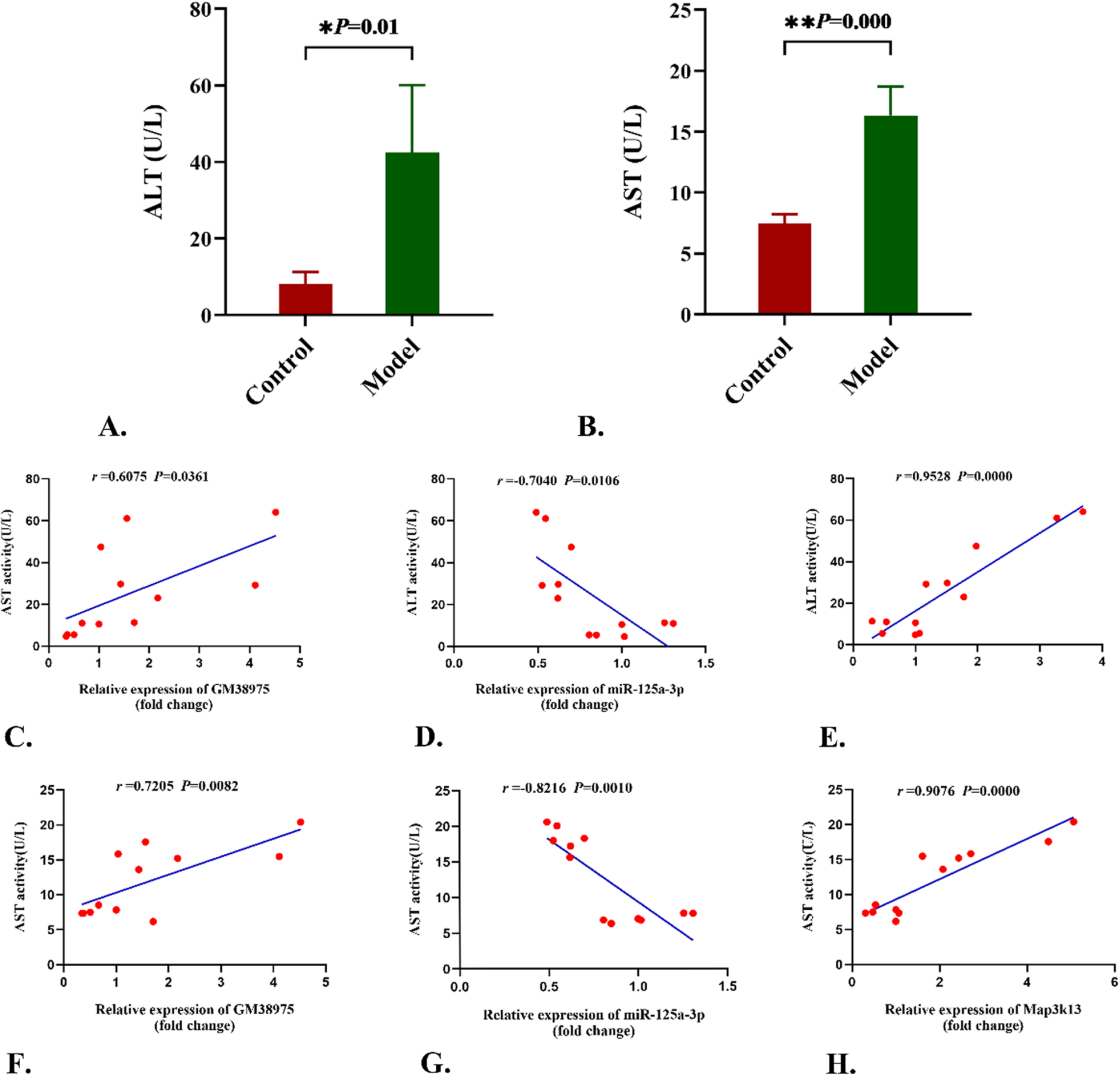
Activity of serum ALT and AST and its correlation with the differentially expressed genes. **A** and **B**. The activity of serum AST and ALT remarkably increased in model group (*P < 0.05, ** *P* < 0.01). C-H. The correlations between the activity of serum AST and ALT and the *Gm38975*, *Map3k13*, *mmu-miR-125a-3p* transcriptional expression levels

As shown in Fig. 7A and B, compared with normal group, the activity of serum ALT and AST drastically increased in response to Con A administration in mice (*P* < 0.01). The activity of serum ALT and AST were positively correlated to the GM38975 and Map3k13 transcriptional level expression, while negatively correlated to the mmu-miR-125a-3p transcriptional level expression (Fig. 7C–H).

### Levels of MDA and NO

**Fig. 8:**
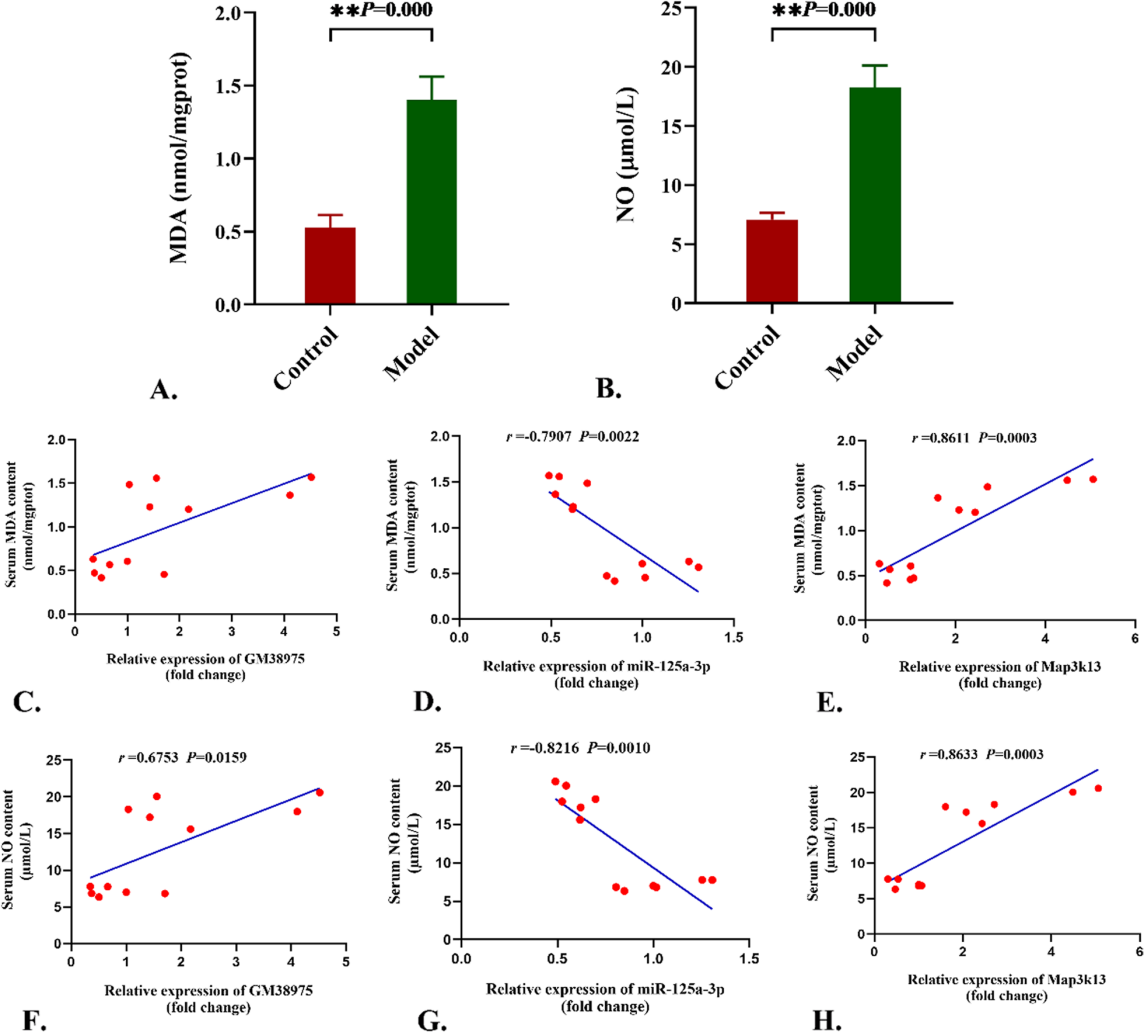
Levels of MDA and NO and their correlation with the differentially expressed genes. **A** and **B**. The content of MDA and NO significantly elevated in the model group (*P < 0.05, **P < 0.01). C-H. The correlations between the content of MDA and NO and the Gm38975, Map3k13, mmu-miR-125a-3p transcriptional expression levels

MDA is one of the most important products of membrane lipid peroxidation, while NO plays a vital role in the oxidative stress response as a kind of reactive nitrogen species (RNPs). All of them can be known as an indirect indicator for the oxidative stress damage in liver. As exhibited in Fig. 8A and B, the biochemical analysis results showed that the amount of MDA and NO were significantly higher in the liver of model group mice than that in control group (*P* < 0.01). There were positive correlations between the content of MDA or NO and the GM38975 and Map3k13 transcriptional level expressions, as well as negative correlations between the levels of these two indicators for oxidative stress damage and the mmu-miR-125a-3p transcriptional level expression (Fig. 8C–H).

## Discussion

Against the background of that the aberrant expression of miRNA has been more and more frequently reported in autoimmune diseases [31, 32], particularly in AIH [33, 34], a new miRNA-associated perspective of determining the pathogenesis of AIH is sorely required. Since the hypothesis proposed in 2011, it has been believed that the perturbation of ceRNA networks has consequences for a variety of diseases. However, on the flip side, it also presents opportunities for explaining disease processes and new therapies, although the understanding on this field is still in their infancy. Except the ceRNA researches involved in tumor genesis, invasion and metastasis being in the ascendant nowadays [35–37], a growing number of studies have been provided evidences that the pathogenesis of autoimmune diseases have some relationship with the disturbance of this certain miRNA sponge mechanism [38, 39]. It has been proven that lncRNA PVT1 has the potency to hinder the progression of rheumatoid arthritis (RA). Fibroblast-like synoviocyte (FLS) is the key cell type for promoting the joint destruction after obtaining an aggressive phenotype via resistance to apoptosis [40]. LncRNA PVT1 can act as ceRNA to induce the apoptosis of FLS through modulating the expression of miR-543-dependent signal peptide-CUB-EGF-like containing protein 2 (SCUBE2) [41]. Meanwhile, a recent high-throughput RNA sequencing (RNA-seq) study related to systemic lupus erythematosus (SLE), another common autoimmune disease, has revealed that lncRNA MIAT and NEAT1 probably play crucial roles in the pathogenesis of SLE by interacting with hsa-miR-145, hsa-miR-17 and hsa-miR-143 as ceRNAs [42]. Moreover, hepatic fibrosis is the usual outcome of AIH, and accumulating reports have indicated that lncRNAs played a significant role in the initiation and progression of hepatic fibrosis by serving as ceRNAs to regulate the expression of target gene [43, 44]. Thus, it is quite necessary to explore the relationship between the ceRNA network and etiopathogenesis of AIH.

Although the profiling of DELs, DEMis and DEMs in the Con A-induced AIH model have been reported in our earlier research [23, 24], the interactions between these differentially expressed genes were still not yet elucidated. In this study, the ceRNA network associated with the occurrence of AIH was constructed for the first time, based on the microarray data mentioned above. Numerous filtered DELs, DEMis, and DEMs, which were all well clustered in the model group, provided the foundation for the construction of ceRNA. The miRNA-mRNA, miRNA-lncRNA and mRNA-lncRNA co-expression pairs were predicted according to the set threshold of |PCC| and *P* value, and the information of MER on the sequences of some lncRNAs and mRNAs were labeled and exhibited in Fig. 2. The predicted sequences of the MERs will help us a lot to explore the interactions between different genes in depth. Based on these predicted co-expression pairs, we established the ceRNA network related to AIH and part of it (top 200 pairs in the order of “ceRNA Score”) was visualized in Fig. 3. Some of the 12 DEMis constructed in this ceRNA network have been reported to be linked to the inflammatory process in liver, such as miR-233 [45, 46], miR-125 [47], miR-486a-5p [48]. These results showed some new insights into the pathogenesis of AIH to us by opening out the intrinsic connections among these aberrantly expressed of miRNAs.

In order to further demonstrated some of the underlying biological consequences of this constructed ceRNA network, GO enrichment analysis was carried out. We found that the DEMs were most significantly involved in the BP category of “inflammatory response” and MF category of “protein binding” (see Fig. 4). As known from the published reports on AIH, disturbance of immunological tolerance was the main reason for the necrosis of liver cells, and increased production of proinflammatory cytokines was one of the typical pathological features in this disease [5, 49]. The result related to the GO term of “inflammatory response” not only emphasized the pathophysiological roles of this immediate defensive reaction to injury in the development of AIH once again, but also revealed the regulation feature of these ceRNAs in the process of inflammation. “Protein binding”, synonyms of “protein amino acid binding” or “glycoprotein binding”, is the GO terms referring to “interacting selectively and non-covalently with any protein or protein complex”. It is well known that the immune responses rely on the selective interactions between receptors and their ligands, and the autoimmune diseases will be triggered once the homeostasis of the interaction relationship is broken [50–52]. The biological features connecting with the GO term of “Protein binding” gave us a new perspective on how these ceRNA functioned in the occurrence of AIH, as well as offered new target and orientation for the therapy of this disorder. In addition, the regulatory process of ceRNA were also closely related with the response to “metal ion binding” (MF, GO:0046872), which had been acknowledged as the notable activities in the early stage of T cell-mediated autoimmune diseases [53]. This result indicated that the potential regulation functions involved in the abnormal activation of autoimmune T cells undertaken by ceRNA may have some relationship with the metal ion metabolism, such as iron deposition [54, 55].

The results of KEGG enrichment showed that 105 signaling pathways were possibly mediated by these ceRNAs, including “MAPK signaling pathway”, “TNF signaling pathway”, “PI3K-Akt signaling pathway” and “Toll-like receptor signaling pathway”. Notwithstanding accumulating reports have confirmed that these pathways indeed played essential roles in the pathological process of inflammation in liver, the exact mechanism for giving rise to the anomaly in these signaling pathways has yet been clarified [56–59]. While reemphasizing the importance of these signaling pathways in the development of AIH, the constructed ceRNA network can throw light on the future study on the dysfunction of these signal pathways form a new “RNA-RNA interaction” perspective. Furthermore, as the most significantly being enriched signaling pathway, “Cytokine-cytokine receptor interaction” pathway was deemed to be crucial intercellular regulator and mobilizer of cells engaged in innate as well as adaptive inflammatory host defenses in liver, and immune injury to liver will be triggered in case of that the homeostasis of the cytokine-cytokine receptor interaction is broken and not able to be restored [60, 61]. Our findings in this study corroborated the specific role of “Cytokine-cytokine receptor interaction” linked to AIH again, and was helpful to provide new therapeutic strategies for this disease by intervening the anomalous interaction between cytokines and their receptors as well.

At last, the expression of GM38975, mmu-miR-125a-3p and Map3k13, one of the potential ceRNA pair, were validated at the level of transcription and translation with qRT-PCR and western-blot assay. Map3k13, also known as leucine zipper bearing kinase (LZK), is one of the main kinase components in the MAPK signal cascade. It can regulate the activity of p38 through phosphorylation and play an important role in the occurrence and development of liver inflammatory diseases or tumors [62, 63]. miR-125a-3p is closely related to the expression regulation of p38. The increased expression of miR-125a-3p can significantly reduce the expression level of p38 and alleviate the inflammatory injury mediated by the latter [64, 65]. In our study, the expression of GM38975 and Map3k13 at transcriptional level were up-regulated, while that of mmu-miR-125a-3p was down-regulated in model group. The elevated expression of Map3k13 at translational level were also detected in model group. Therefore, the aberrant expression of these three genes were supposed to have some relationship with the occurrence of AIH. There were negative correlations between mmu-miR-125a-3p transcriptional level expression and GM38975 or Map3k13 transcriptional level expression. It suggested that these three genes have the potential to form a ceRNA network. Moreover, the expression of GM38975, mmu-miR-125a-3p and Map3k13 at transcriptional level were positively or negatively correlated to the activity of ALT and AST as well as the amount of MDA and NO. These results taken together indicated that after the autoimmune response being triggered by various pathogenic factors (such as Con A), the expression of GM38975 at transcriptional level elevated. The up-regulated GM38975 can “adsorb” mmu-miR-125a-3p competitively with Map3k13 through miR-125a-3p MER in their sequence, thereby weakening the negative regulation of mmu-miR-125a-3p on transcription of Map3k13. The increased expression of Map3k13 can mediate the direct inflammatory liver injury, or activate the oxidative stress pathway. It was signified that as a ceRNA, GM38975 probably cooperates with mir-125a-3p to participate in the regulation of Map3k13 expression, which may be one of the key mechanisms to induce liver inflammatory injury. In-depth study on the above synergistic regulatory mechanisms will help to further reveal the upstream transcriptional regulatory network of “MAPK mediated liver inflammatory injury”. However, the detailed mechanism of regulating the development of AIH need to be further elucidated, because the regulatory networks or mechanisms analyzed in this study are mainly based on bioinformatics predictions, and there is a lack of practical experiments to verify these results. At the same time, this study only involved mice with acute liver injury, which cannot fully cover the pathogenesis of AIH, because some AIH patients present with chronic inflammatory process. Moreover, further studies on clinical samples of AIH patients are needed to performed to illustrate the physiological and pathological processes of AIH in deep.

## Conclusion

In summary, our study in this paper provided the comprehensive landscape of ceRNA network related to AIH for the first time, what is of great significance to reveal the“miRNA sponge” roles of lncRNAs to prevent or promote the harms caused by miRNAs in AIH patients. The potential biological functions of the calculated ceRNA network were significantly annotated in the processes of “inflammatory response” and “protein binding”, and the signaling pathways to which they were critically contributed were “Cytokine-cytokine receptor interaction”, “MAPK signaling pathway” and “TNF signaling pathway”. In addition, according to the results of animal experiment, GM38975/miR-125a-3p/Map3k13 have the potential to form a ceRNA network to regulate the “MAPK mediated liver inflammatory injury”. There is no doubt that a much deeper study on the mechanism of ceRNA is needed to provide further insights into the comprehension of AIH with great promise to discover novel therapeutic targets for this world-wide autoimmune disease.

## Supplementary Information

**Additional file 1**. Supplementary Figures and Tables.

## Data Availability

The datasets analysed during the current study are available for download in the Gene Expression Omnibus (GEO) database repository, https://www.ncbi.nlm.nih.gov/geo/”.

## Abbreviations

AIH: Autoimmune hepatitis
ceRNA: Competing endogenous RNA
mRNA: Messenger RNA
ncRNA: Non-coding RNA
MRE: MicroRNA response element
miRNA: MicroRNA
lncRNA: Long non-coding RNA
Con A: Concanavalin A
DEL: Differently expressed lncRNA
DEMi: Differently expressed miRNA
DEM: Differently expressed mRNA
MDA: Malondialdehyde
ALT: Alanine aminotransferase
AST: Aspartate aminotransferase
NO: Nitric oxide
SDS-PAGE: SDS–polyacrylamide gel electrophoresis
PCA: Principal component analysis
PCC: Pearson correlation coefficient
GO: Gene ontology
CC: Cellular components
MF: Molecular functions
BP: Biological processes
FDR: False discovery rate
KEGG: Kyoto Encyclopedia of Genes and Genomes
SPF: Specific pathogen free
NCBI: National Center of Biotechnology Information
DEGs: Differently expressed genes
FC: Fold change
SD: Standard deviation
RA: Rheumatoid arthritis
FLS: Fibroblast-like synoviocyte
SCUBE2: Signal peptide-CUB-EGF-like containing protein 2
RNA-seq: RNA sequencing
SLE: Systemic lupus erythematosus
LZK: Leucine zipper bearing kinase
