# Supplementary material for: Construction and function analysis of the LncRNA-miRNA-mRNA competing endogenous RNA network in autoimmune hepatitis
Source: BMC Med Genomics. 2022 Dec 25;15:270. doi: 10.1186/s12920-022-01416-4 (PMC9790135; doi:10.1186/s12920-022-01416-4)
Supplement: Supplementary file 1 — Additional file 1. Supplementary Figures and Tables. [file 12920_2022_1416_MOESM1_ESM.pdf]

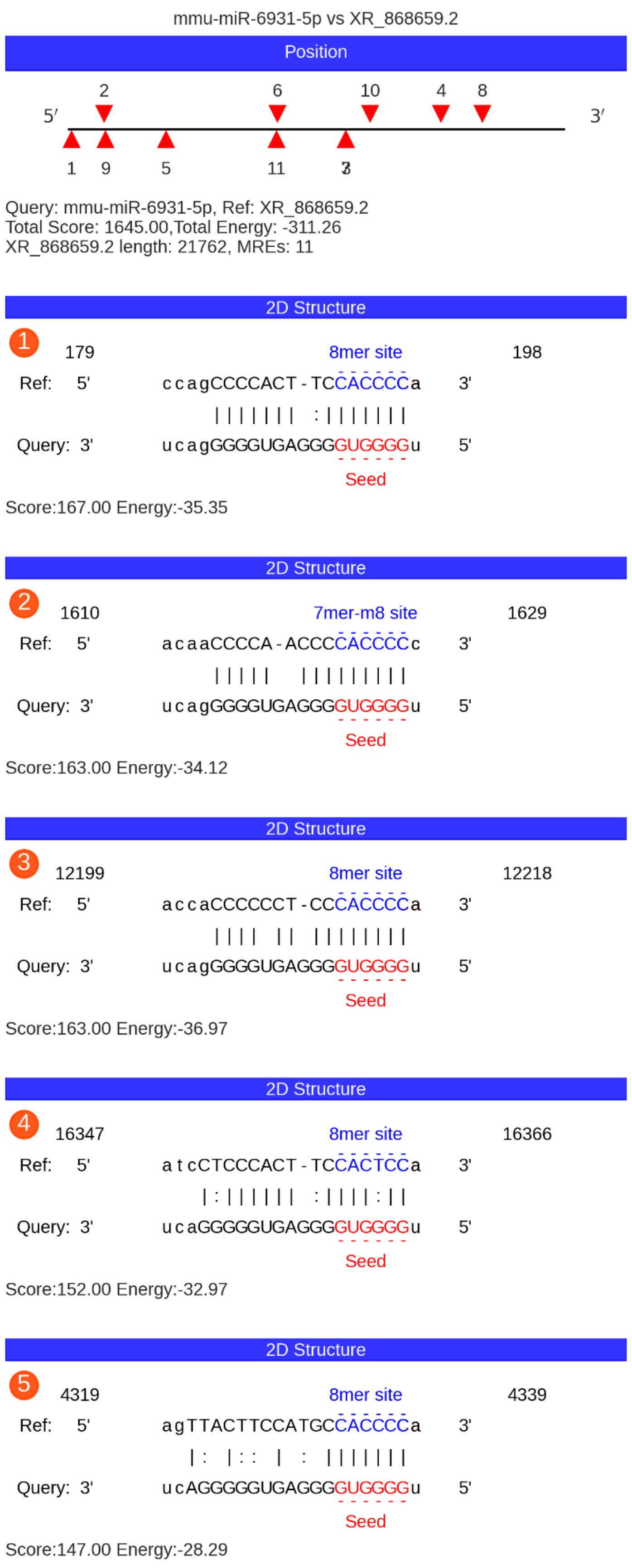

A: mmu-miR-6931-5p vs XR_868659.2

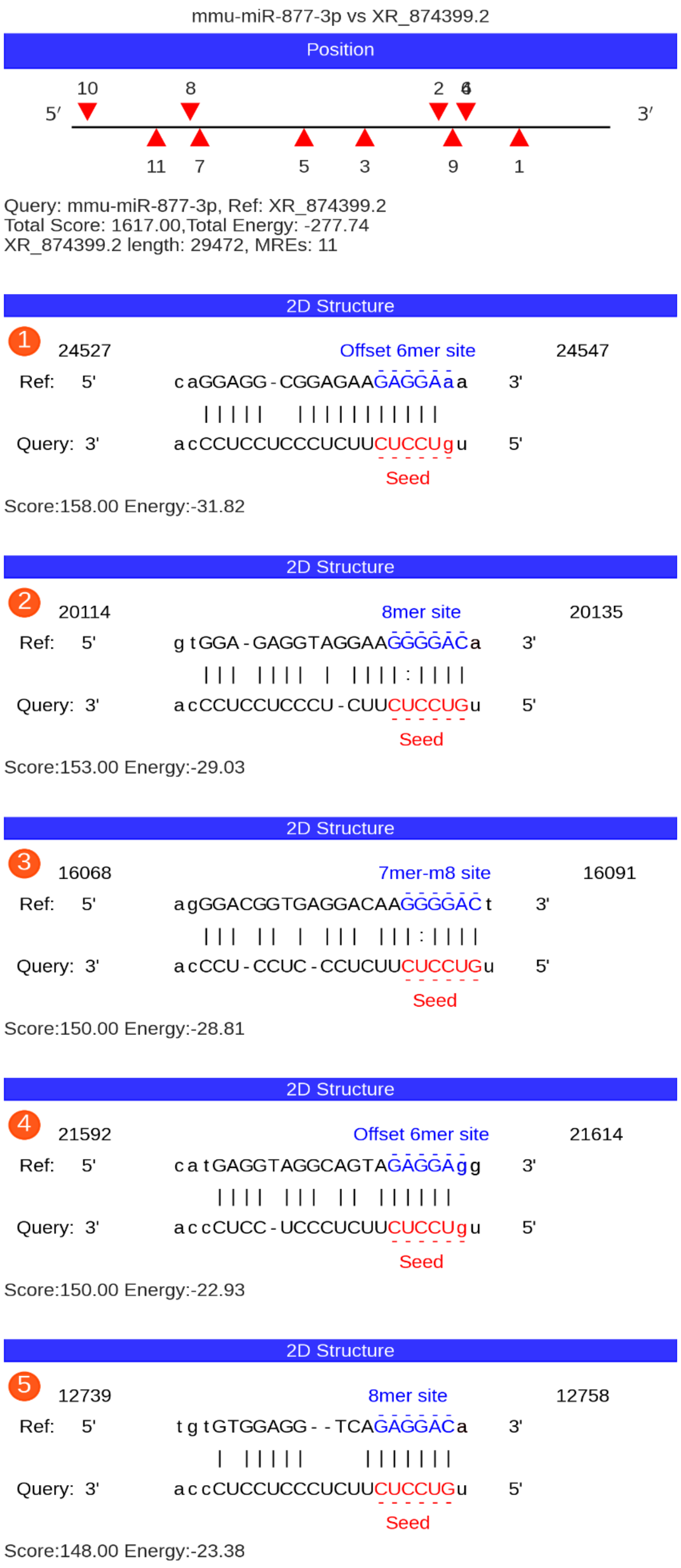

B: miR-877-3p vs XR_874399.2

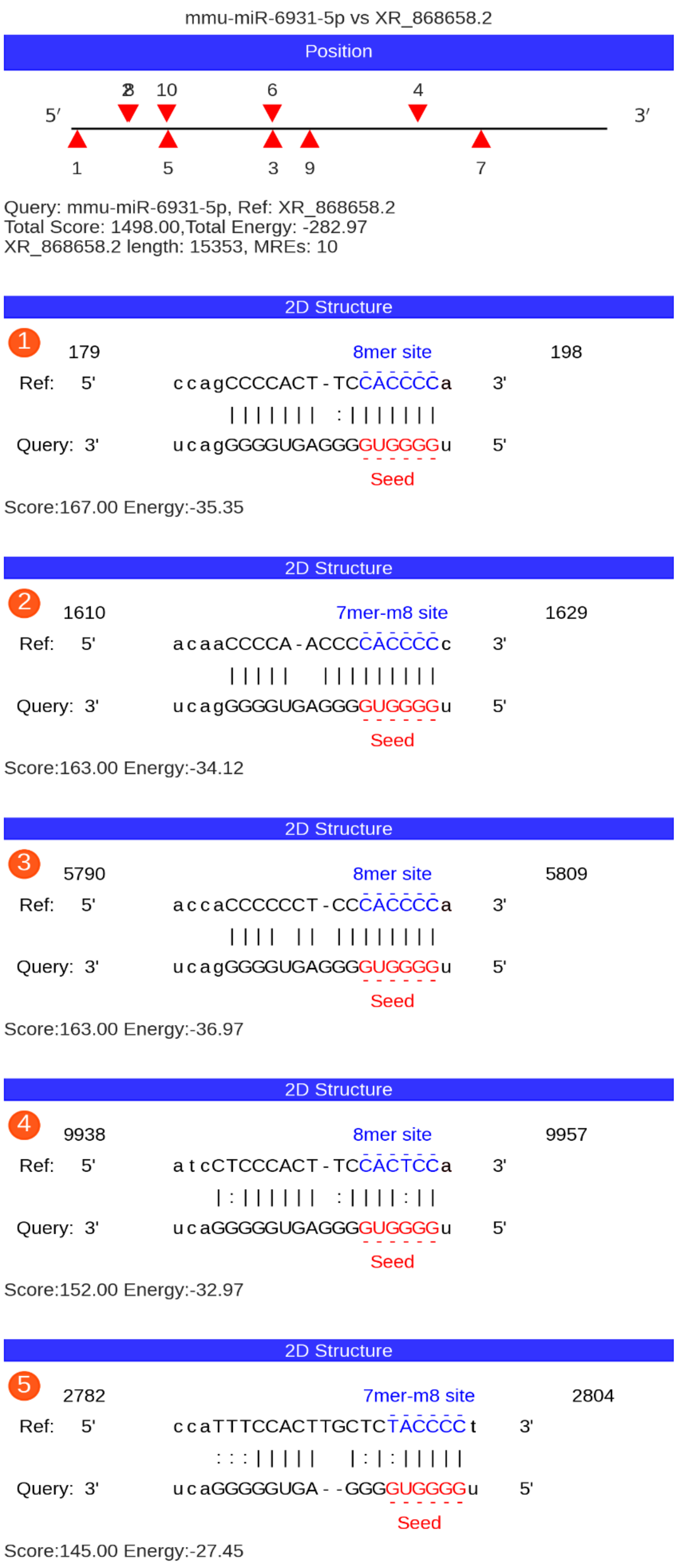

C: miR-6931-5p vs XR_868658.2

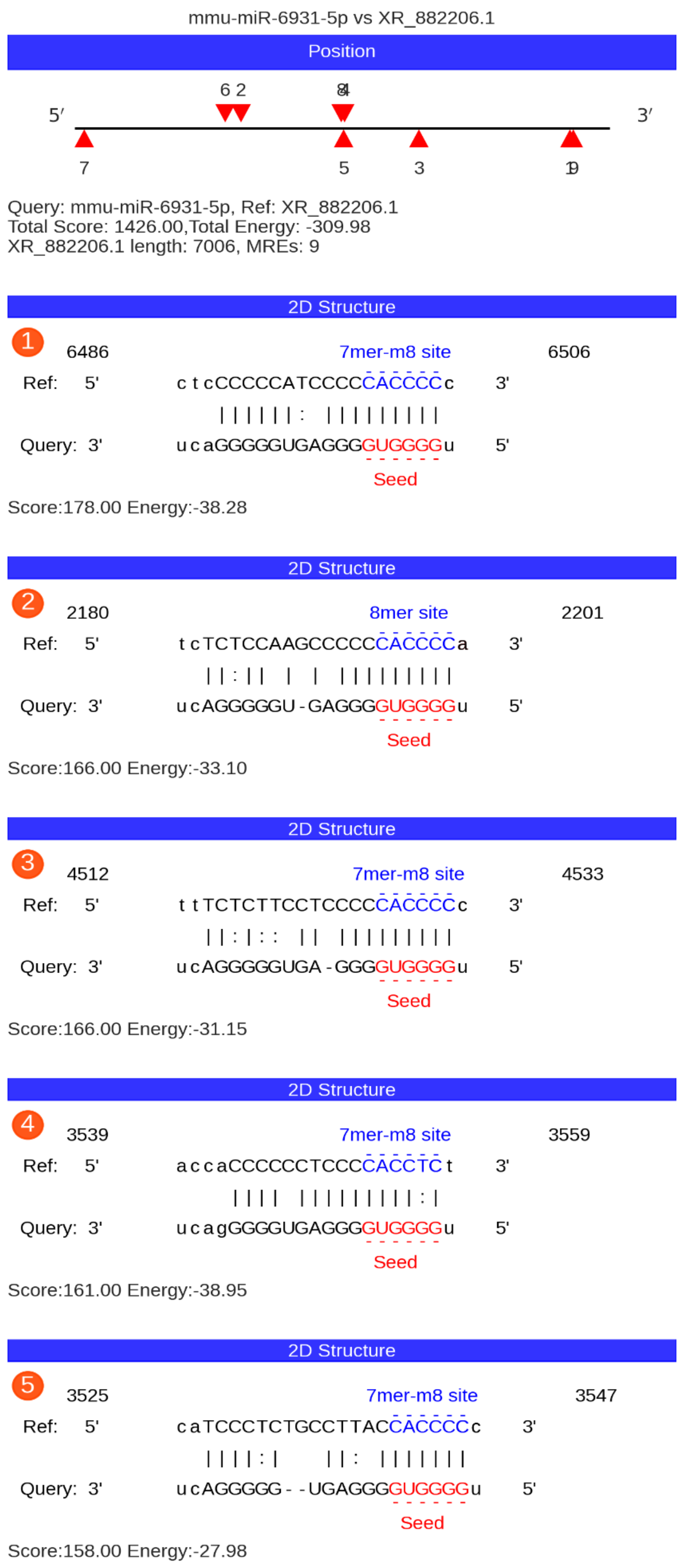

D: mmu-miR-6931-5p vs XR_882206.1

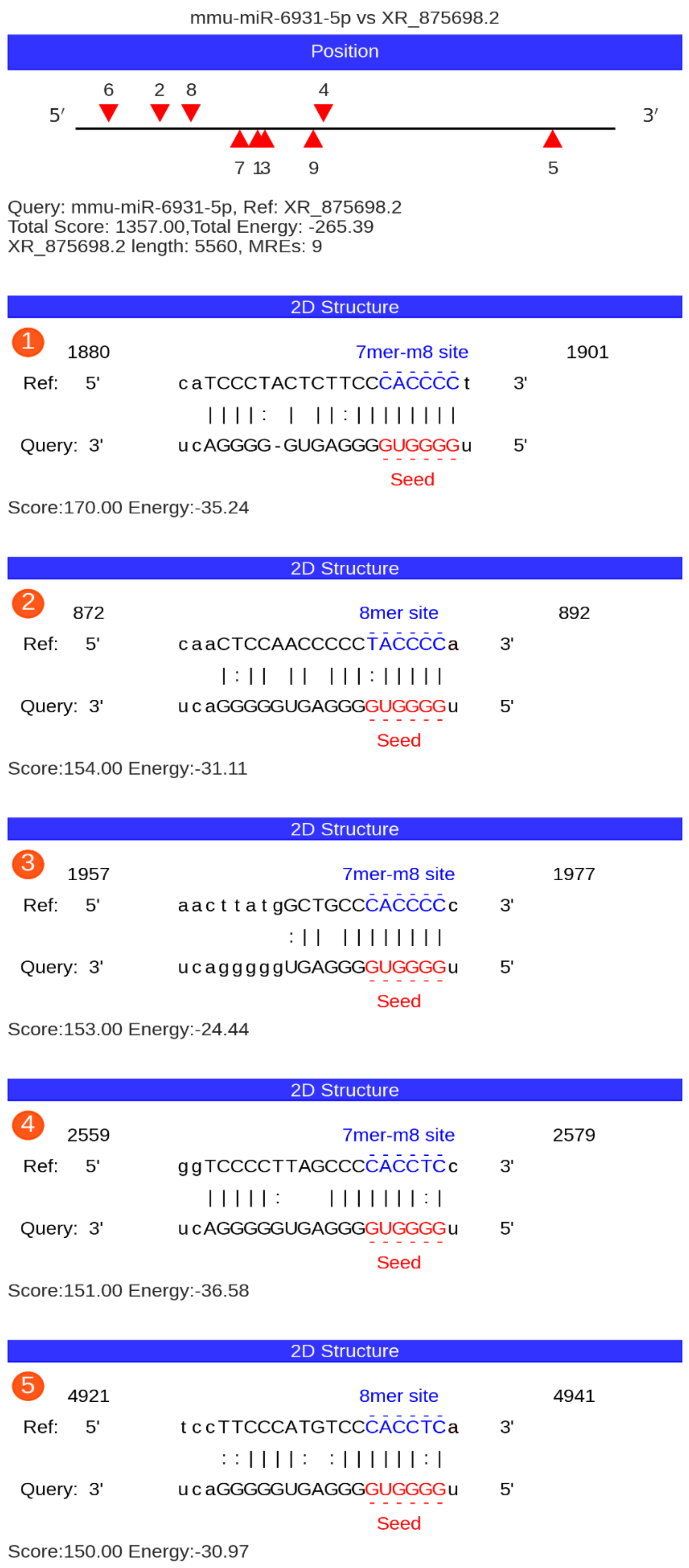

E: mmu-miR-6931-5p vs XR_875698.2

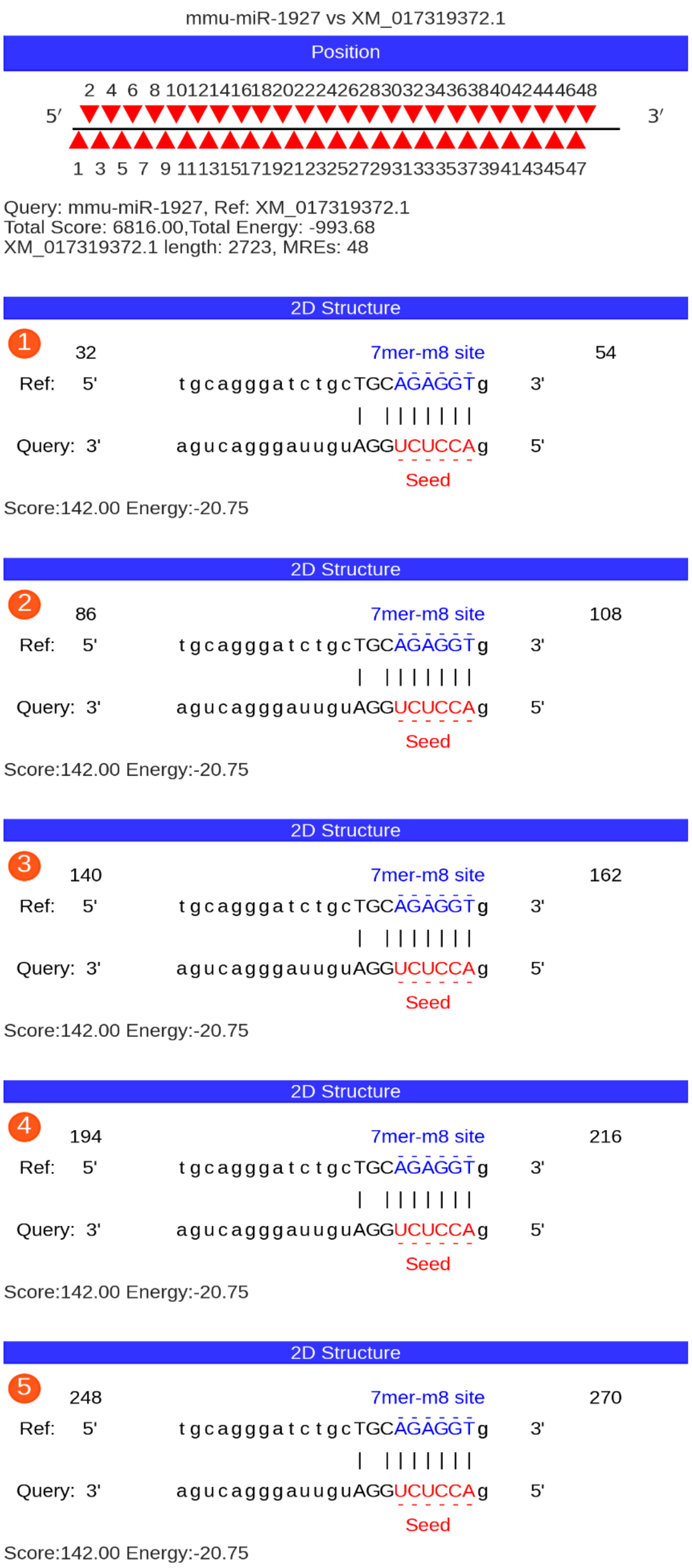

F: mmu-miR-1927 vs XM_017319372.1

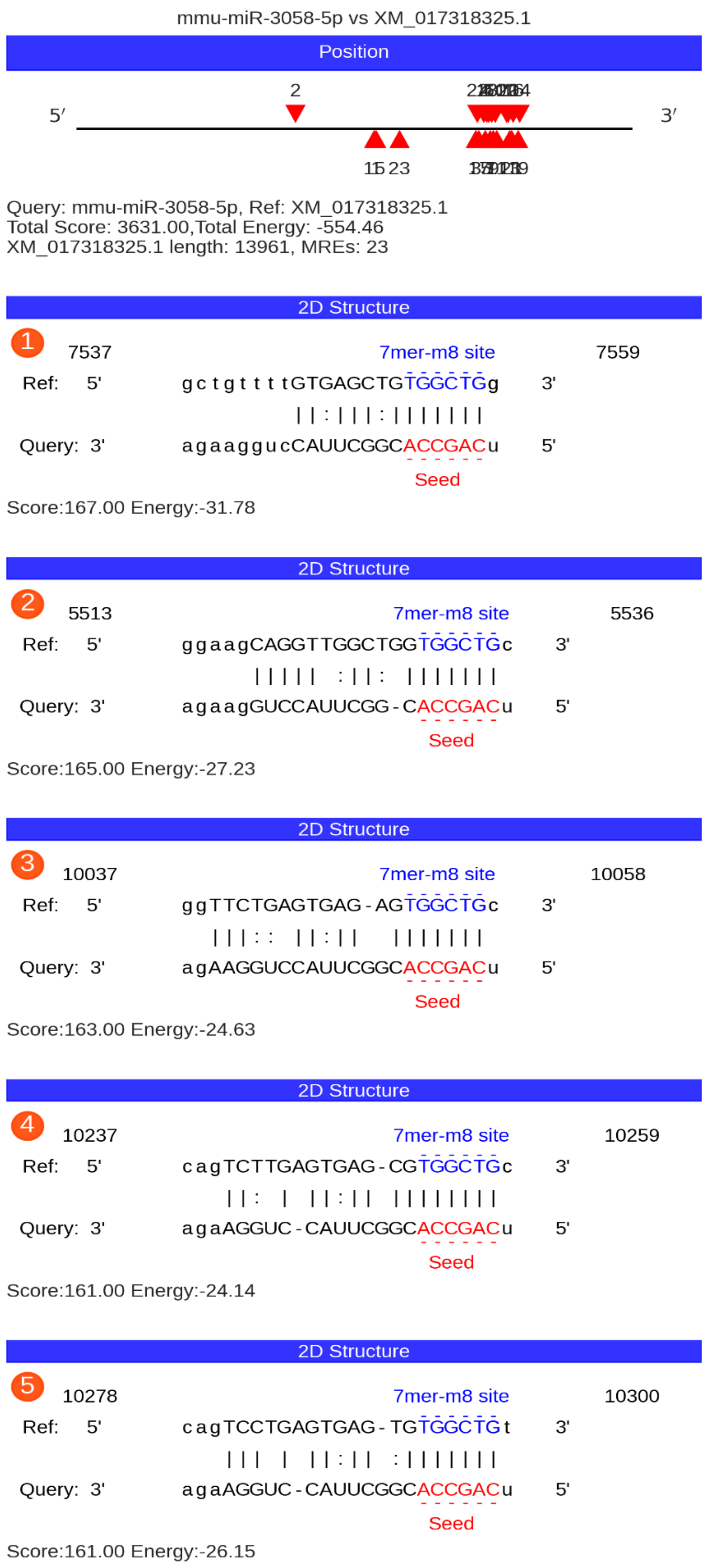

G: mmu-miR-3058-5p vs XM_017318325.1

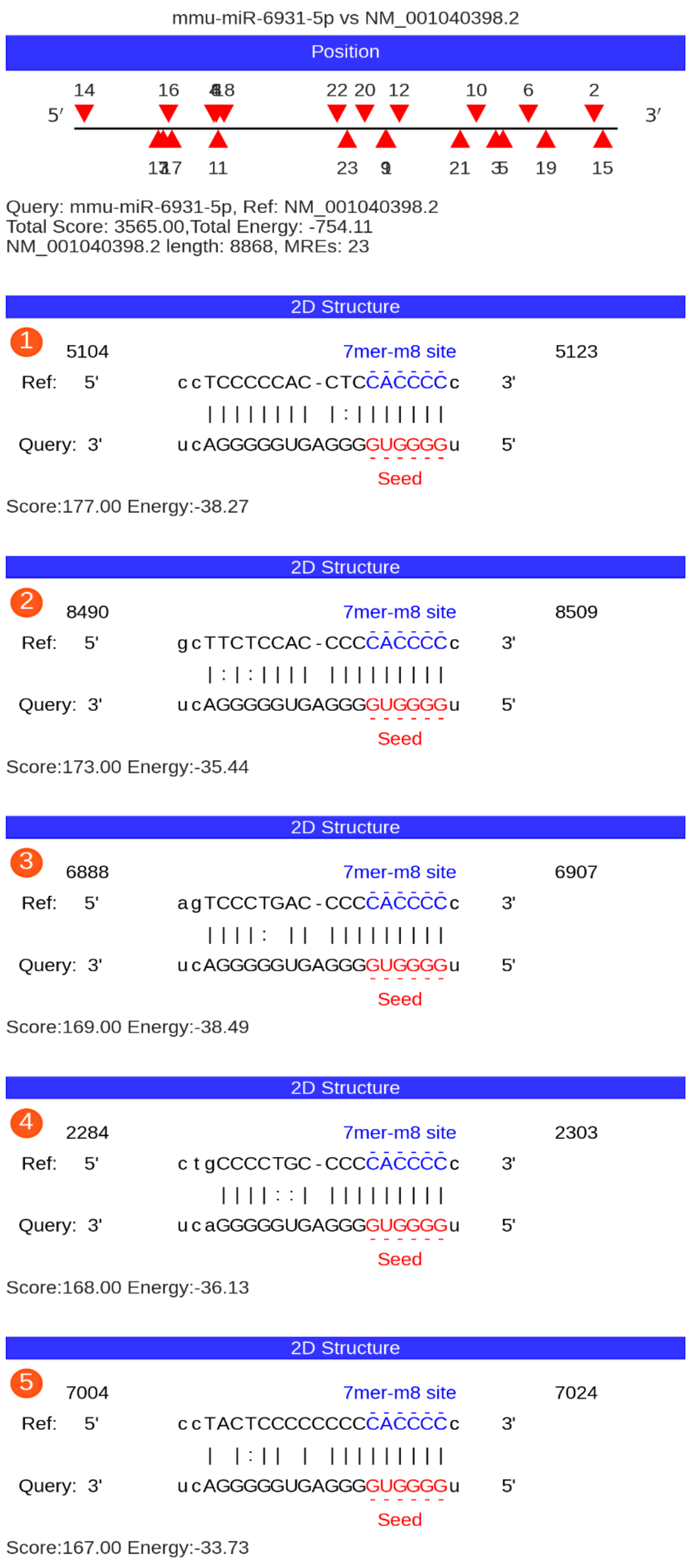

H: mmu-miR-6931-5p vs NM_001040398.2

mmu-miR-6931-5p vs NM_175022.2

Position

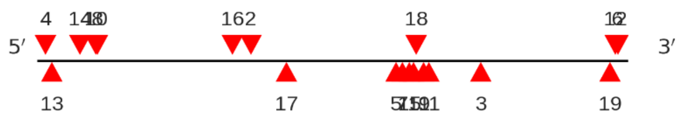

Query: mmu-miR-6931-5p, Ref: NM_175022.2
Total Score: 2828.00,Total Energy: -570.41
NM_175022.2 length: 6879, MREs: 19

2D Structure

```
1   4384                      7mer-m8 site          4403
Ref:    5'   ccaCCTCCAC-CCCCACCCCc      3'
                 ||:||||  |||||||||
Query:  3'   ucaGGGGGUGAGGGGUGGGGu      5'
                             Seed
```

Score:172.00 Energy:-38.23

2D Structure

```
2   2491                      7mer-m8 site          2510
Ref:    5'   cagCCGCCGC-CCCCACCCCc      3'
                 ||  ||:|  |||||||||
Query:  3'   ucaGGGGGUGAGGGGUGGGGu      5'
                             Seed
```

Score:164.00 Energy:-35.44

2D Structure

```
3   5170                      7mer-m8 site          5189
Ref:    5'   gagaagCCA-TCCCCACCCCg      3'
                    |||  |||||||||||
Query:  3'   ucagggGGUGAGGGGUGGGGu      5'
                             Seed
```

Score:161.00 Energy:-31.14

2D Structure

```
4   101                    Offset 6mer site         121
Ref:    5'   gcTCCCGGACTTCCCACCCag      3'
               ||||   |||:|||||||
Query:  3'   ucAGGGGGUGAGGGGUGGGgu      5'
                             Seed
```

Score:155.00 Energy:-32.98

2D Structure

```
5   4182                      7mer-m8 site          4202
Ref:    5'   ggcCCTGGACGACCCACCCCt      3'
                 ||:   ||   ||||||||
Query:  3'   ucaGGGGGUGAGGGGUGGGGu      5'
                             Seed
```

Score:154.00 Energy:-28.41

I: mmu-miR-6931-5p vs NM_175022.2

mmu-miR-193b-3p vs NM_144848.2

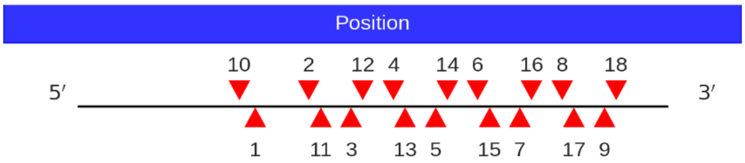

Query: mmu-miR-193b-3p, Ref: NM_144848.2
Total Score: 2565.00,Total Energy: -428.25
NM_144848.2 length: 21627, MREs: 18

2D Structure

```
1   6514                Offset 6mer site      6535
Ref:   5'   tagGGAAGTTTGCAGGCCAGac    3'
               || |  ||||  ||||||
Query: 3'   ucgCCCUGAAACACCCGGUCaa    5'
                            Seed
```

Score:143.00 Energy:-22.45

2D Structure

```
2   8488                Offset 6mer site      8509
Ref:   5'   cggGCGACTTCCAGGGCCAGag    3'
               |  |||||   |||||||
Query: 3'   ucgCCCUGAAACACCCGGUCaa    5'
                            Seed
```

Score:143.00 Energy:-24.68

2D Structure

```
3   10033               Offset 6mer site      10054
Ref:   5'   cggGCGACTTCCAGGGCCAGag    3'
               |  |||||   |||||||
Query: 3'   ucgCCCUGAAACACCCGGUCaa    5'
                            Seed
```

Score:143.00 Energy:-24.68

2D Structure

```
4   11578               Offset 6mer site      11599
Ref:   5'   cggGCGACTTCCAGGGCCAGag    3'
               |  |||||   |||||||
Query: 3'   ucgCCCUGAAACACCCGGUCaa    5'
                            Seed
```

Score:143.00 Energy:-24.68

2D Structure

```
5   13123               Offset 6mer site      13144
Ref:   5'   cggGCGACTTCCAGGGCCAGag    3'
               |  |||||   |||||||
Query: 3'   ucgCCCUGAAACACCCGGUCaa    5'
                            Seed
```

Score:143.00 Energy:-24.68

J: mmu-miR-193b-3p vs NM_144848.2

Supplementary Fig 1. Sequence combination of top 5 pairs of miRNA-mRNA and miRNA-lncRNA in descending order of Total Score. At the top of each figure is the schematic diagram of the sequence of lncRNA or mRNA binding to miRNA. The red triangle represented the MRE and was sorted with Arabic numerals according to the Total Score of each MRE in descending order. The “seed region” of the top 5 MRE in descending order of Total Score were shown below the schematic diagram. A: mmu-miR-6931-5p vs XR_868659.2, B: miR-877-3p vs XR_874399.2, C: miR-6931-5p vs XR_868658.2, D: mmu-miR-6931-5p vs XR_882206.1, E: mmu-miR-6931-5p vs XR_875698.2, F: mmu-miR-1927 vs XM_017319372.1, G: mmu-miR-3058-5p vs XM_017318325.1, H: mmu-miR-6931-5p vs NM_001040398.2, I: mmu-miR-6931-5p vs NM_175022.2, J: mmu-miR-193b-3p vs NM_144848.2

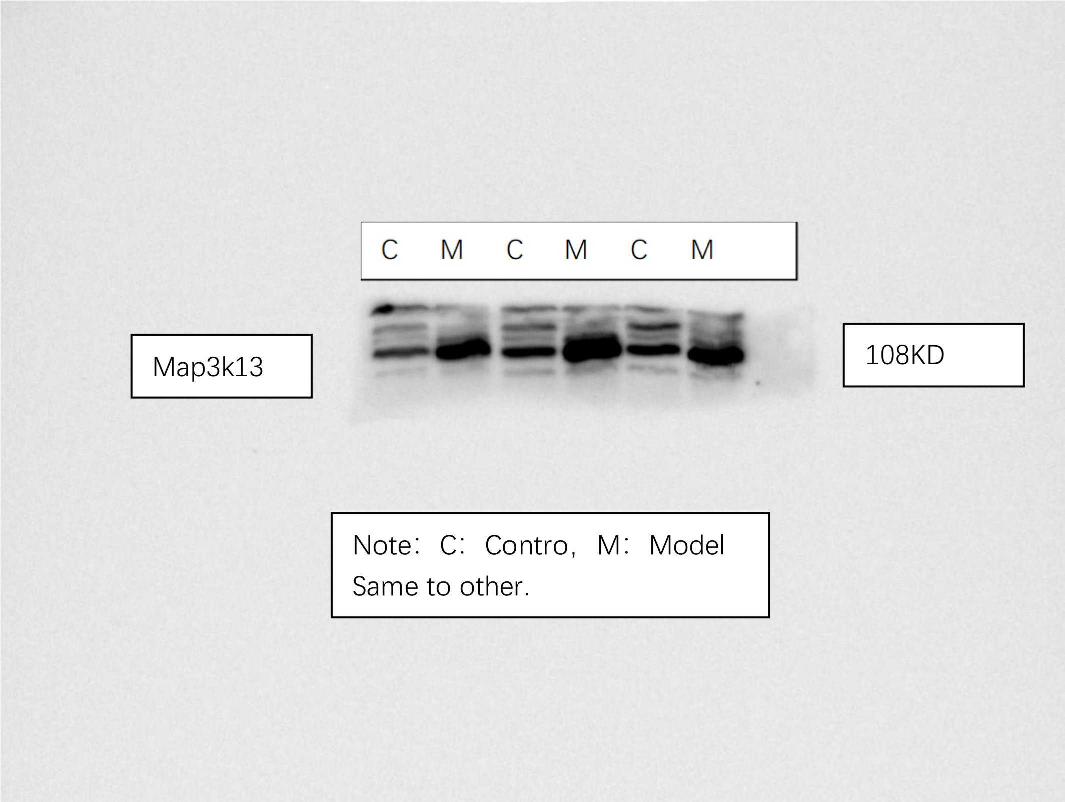
C M C M C M
Map3k13
108KD
Note: C: Contro, M: Model
Same to other.

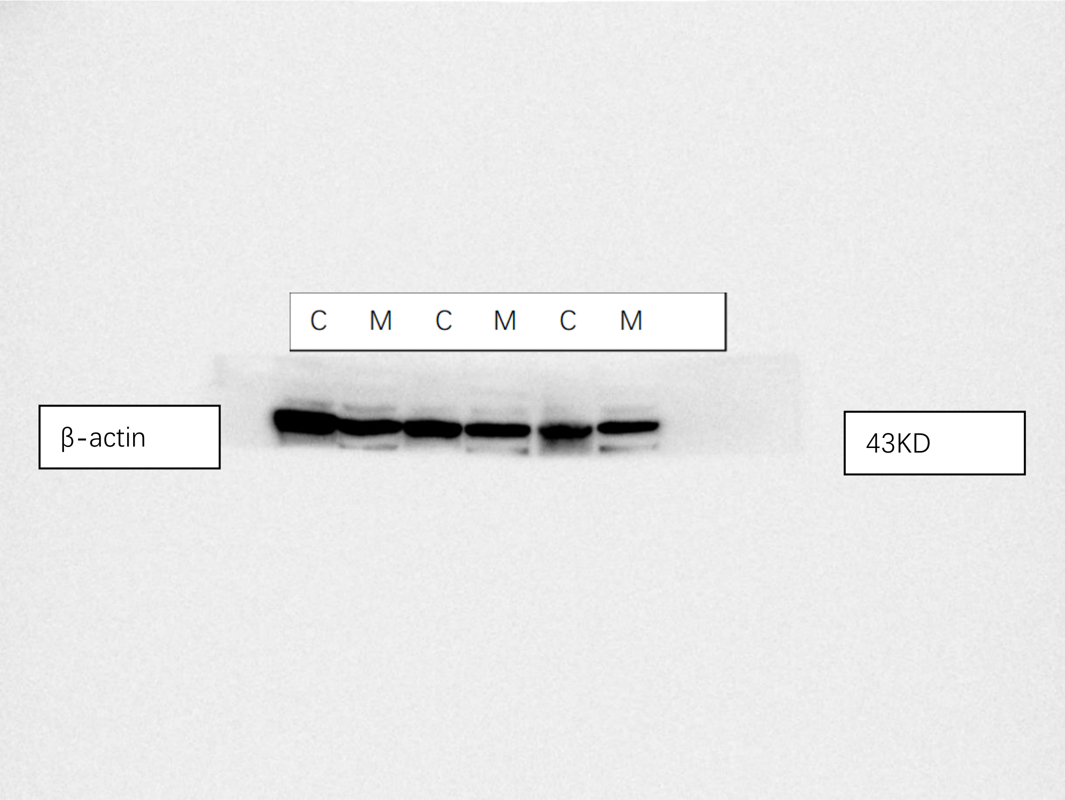
C M C M C M
β-actin
43KD

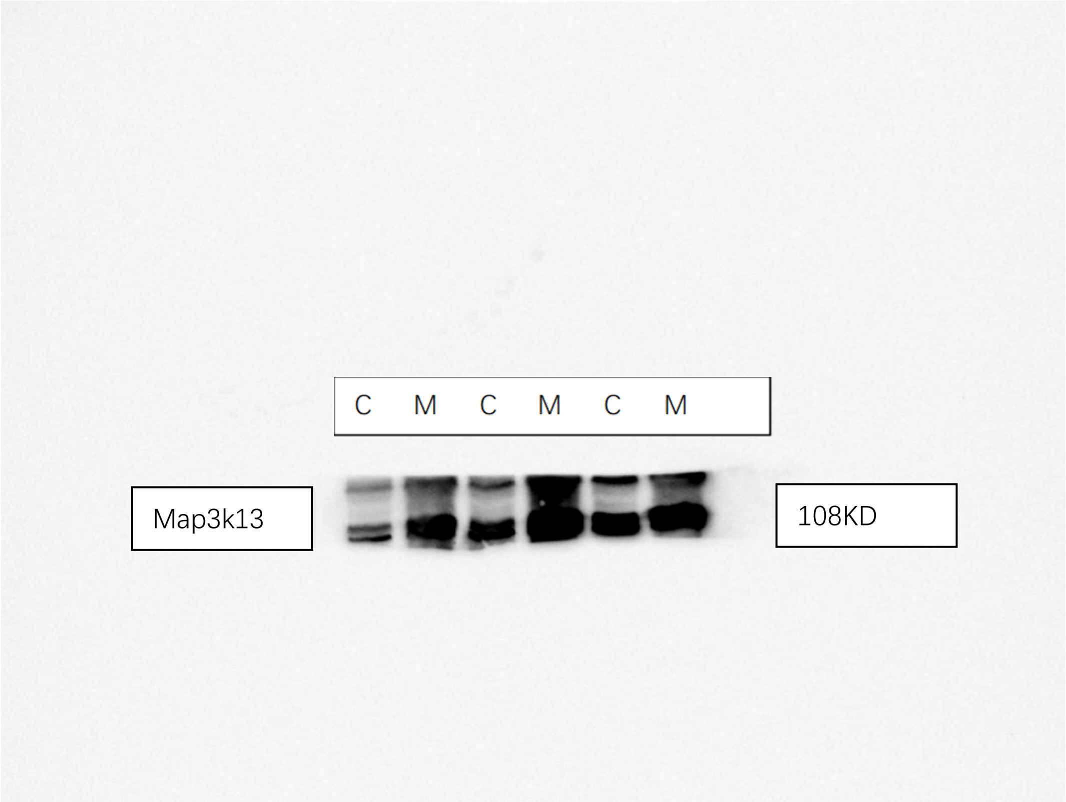
C M C M C M
Map3k13
108KD

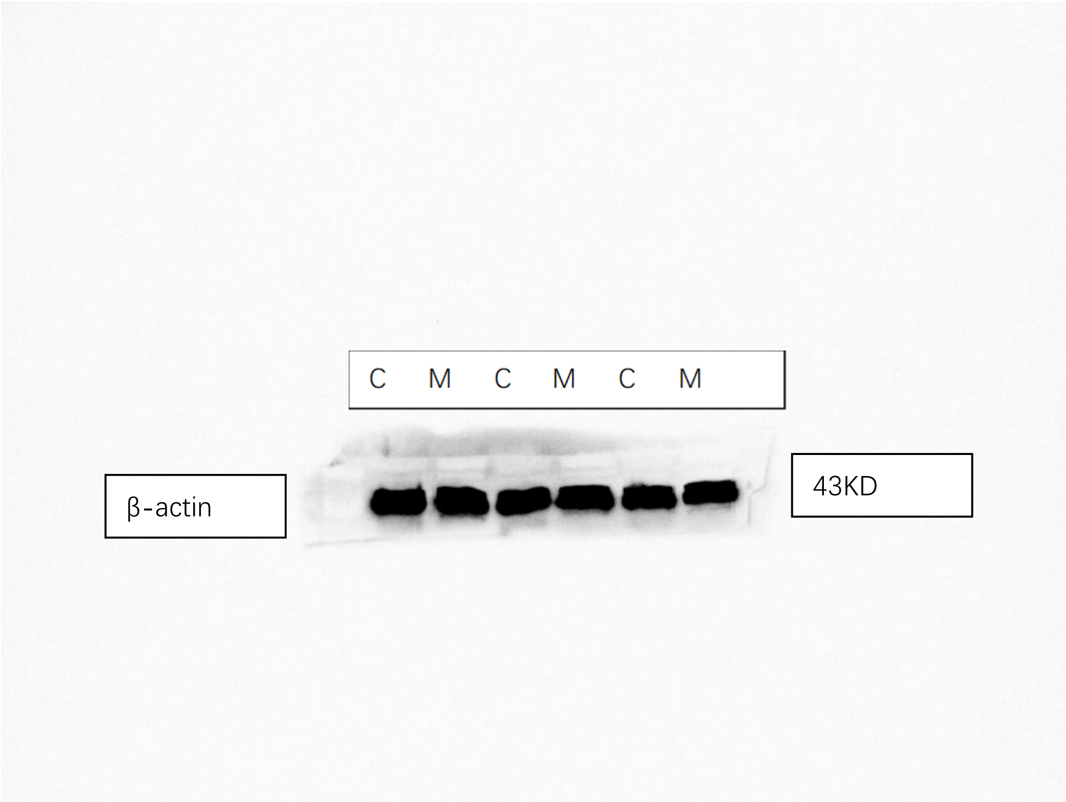
C M C M C M
β-actin
43KD

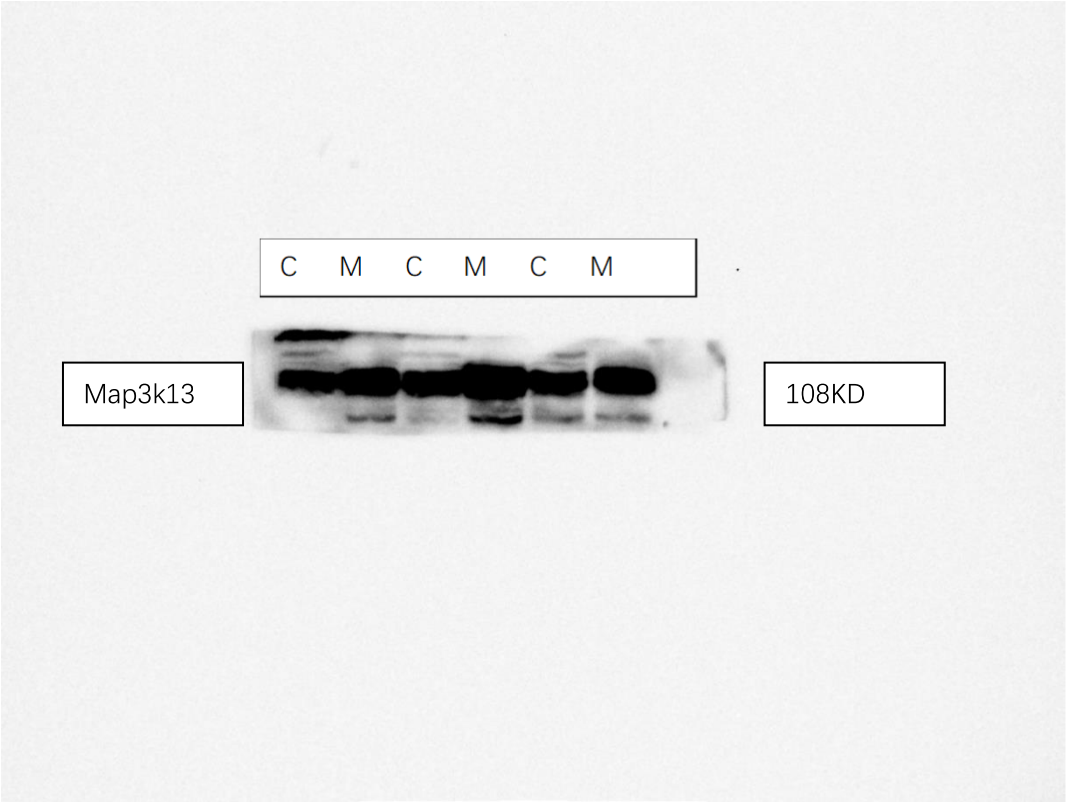
C M C M C M
Map3k13
108KD

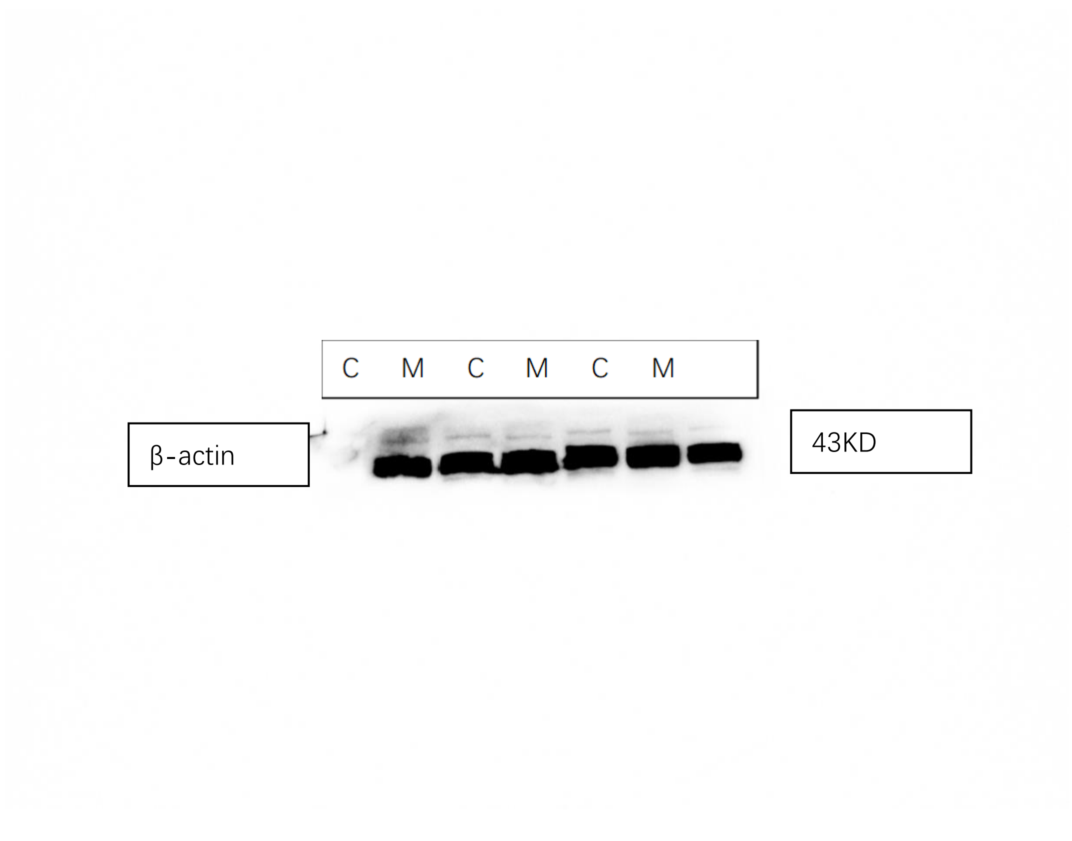
C M C M C M
β-actin
43KD

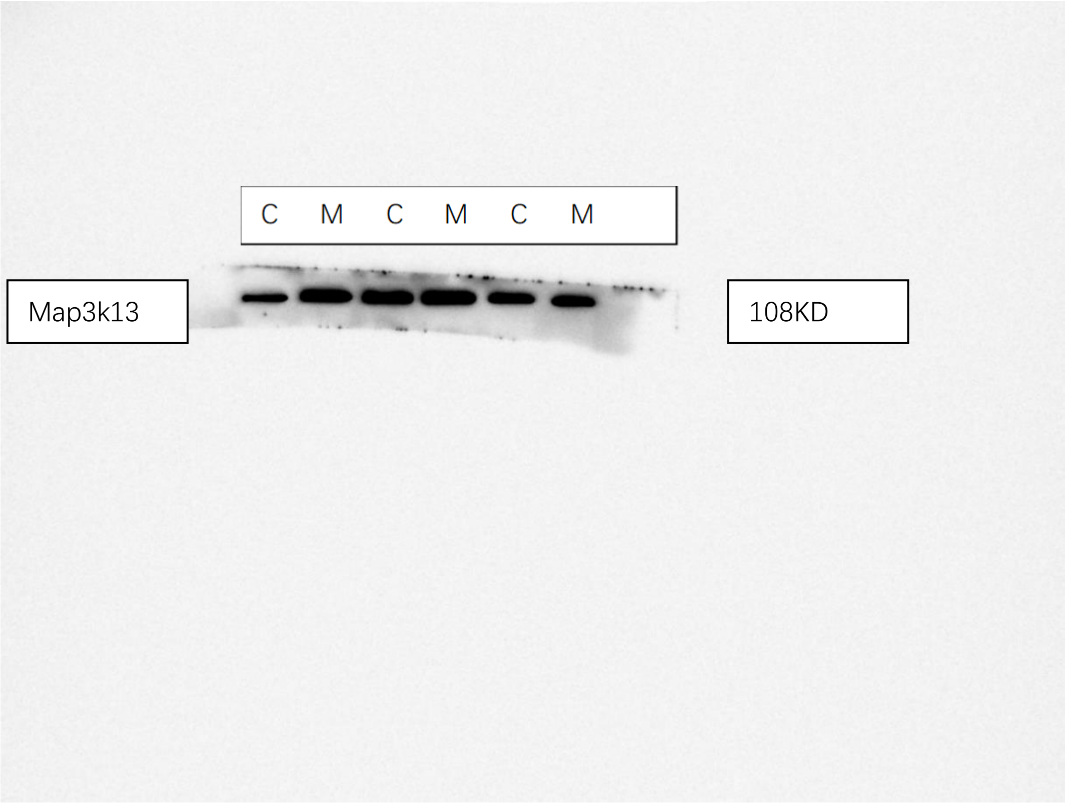
C M C M C M
Map3k13
108KD

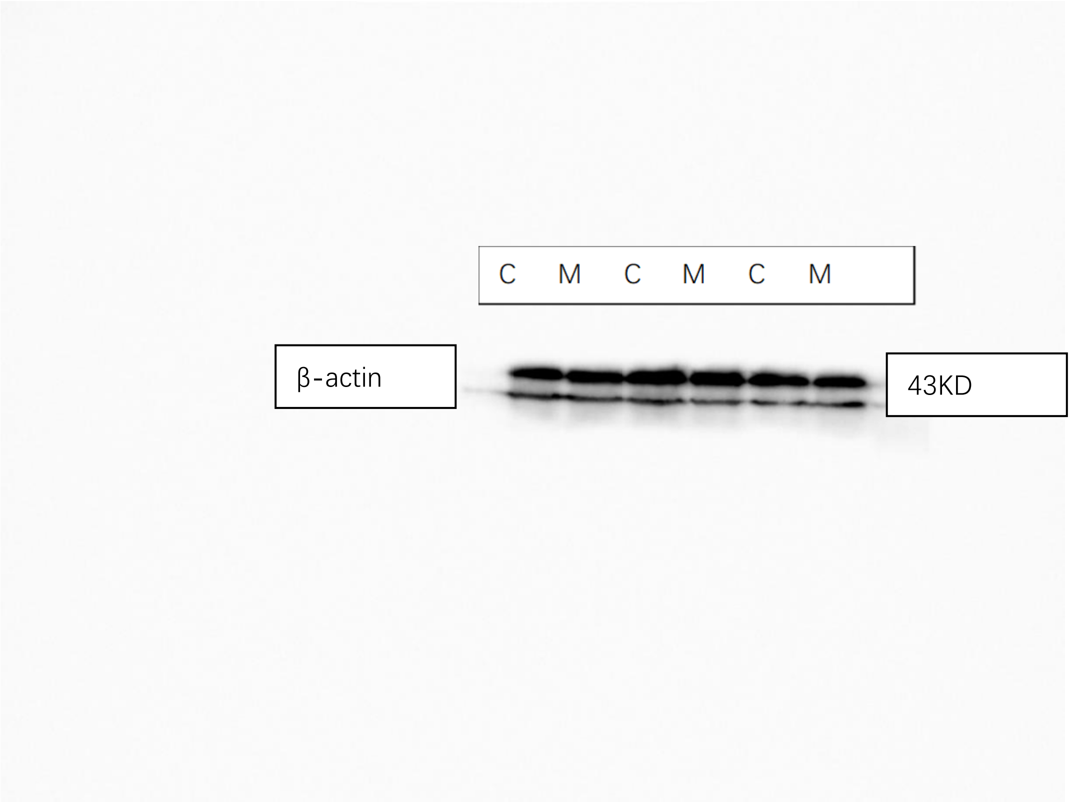
C M C M C M
β-actin
43KD

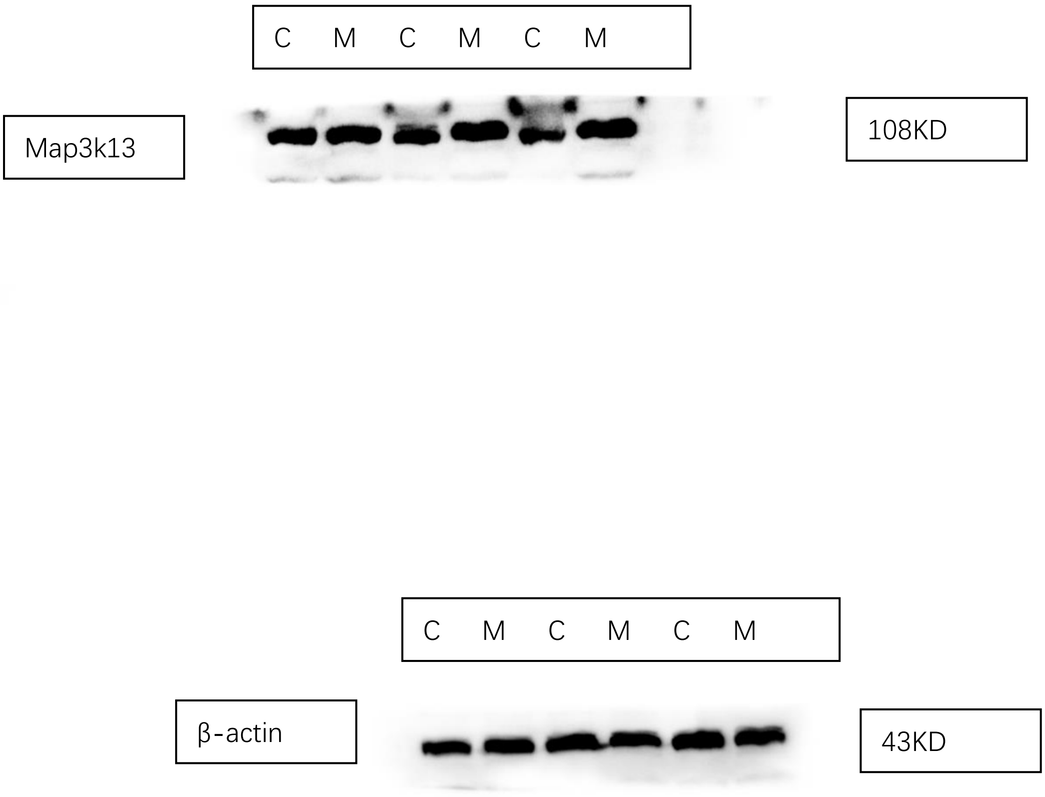
C M C M C M
Map3k13
108KD
C M C M C M
β-actin
43KD

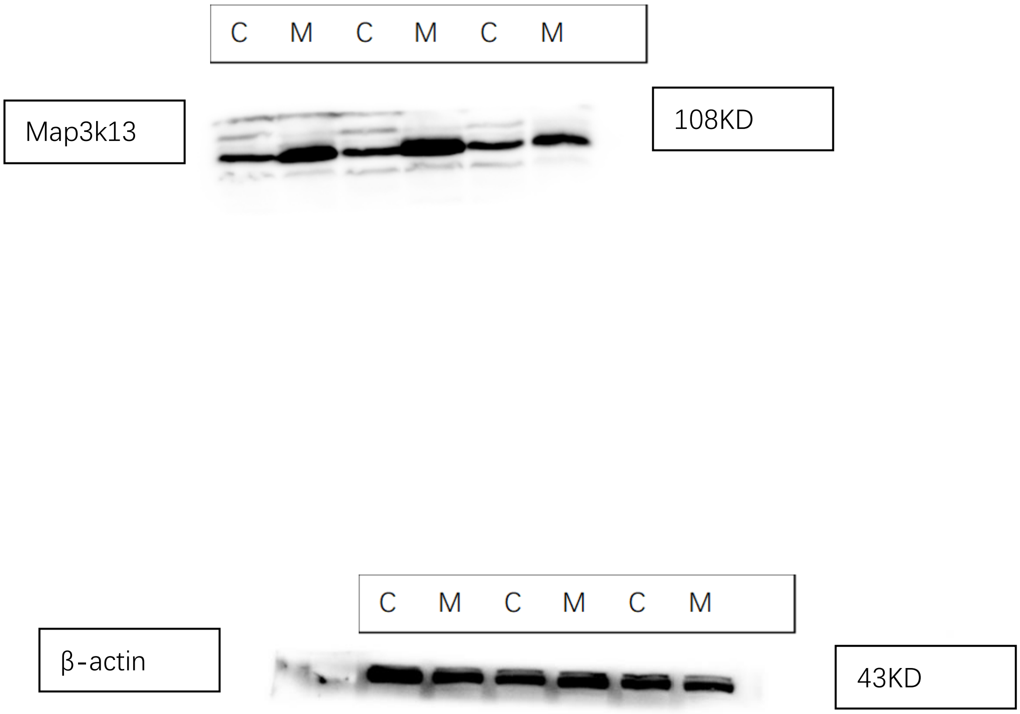

Supplementary Fig 2. All the WB blots of Map3k13 and β-actin protein. The target bands were cropped according to the different molecular weights of the protein prior to hybridization with antibodies.

Supplementary Table 1. Methods related to analysis.

Methods	What To Solve	Ref. or website
miranda V3.3a	predicting the regulatory relationships between miRNA-lncRNA and miRNA-mRNA	http://www.bioinformatics.com.cn/local_miranda_miRNA_target_prediction_120
MuTATE	calculating the ceRNA score	[25]
hypergeometric distribution algorithm	calculating the probability of sharing same miRNAs among ceRNA pairs	[26]
Cytoscape	visualizing the ceRNA network	http://cytoscape.org/
Gene ontology	revealing the characteristics of DEMs involved in the ceRNA network	http://www.r-project.org/
KEGG	revealing the underlying biological activities of these DEMs	https://www.genome.jp/kegg/

Supplementary Table 2. Antibodies for western blotting.

Antibodies	Manufacture	Catalog number	molecular weight	Dilution
Mapk3k13 Rabbit pab	Abclonal	A10189	108kDa	1:1000
Anti-β-Actin Mouse Monoclonal Antibody(1C7)	Abbkine	A01010	43kDa	1:1000
HRP,Goat Anti-Rabbit IgG	Abbkine	A21020		1:1000

Supplementary Table 3. Features of differently expressed genes selected for qRT-PCR validation.

Genes	Accession	Chromosome	FC	*P* value	Regulation
GM38975	XR_001780316.1	chr11	6.08	6.72E-03	up
mmu-miR-125a-3p	MIMAT0004528	chr2	6.18	3.67E-02	down
Map3k13	NM_172821.3	chr16	4.24	9.00E-04	up

Supplementary Table 4. Sequences of primers

Genes	Primers
GM38975	F: 5' GAGGTTAGAGTGCCTGTGAGTGA 3'
	R: 5' GTGGTGGGGTTGATGGGA 3'
mmu-miR-125a-3p	RT: 5' CTCAACTGGTGTCGTGGAGTCGGCAATTCAGTTGAGGGCTCCC 3'
	F: 5' ACACTCCAGCTGGGACAGGTGAGGTTCTT 3'
	R: 5' TGGTGTCGTGGAGTCG 3'
Map3k13	F: 5' GTTGACCAGTGTAAGCGAGGAT 3'
	R: 5' GCCTGACCTGCTGAACTGAA 3'
GAPDH	F: 5' GGTTGTCTCCTGCGACTTCA 3'
	R: 5' TGGTCCAGGGTTTCTTACTCC 3'
U6	F: 5' CTCGCTTCGGCAGCACA 3'
	R: 5' AACGCTTCACGAATTTGCGT 3'
